# Bacteria in Honeybee Crops Are Decoupled from Those in Floral Nectar and Bee Mouths

**DOI:** 10.1007/s00248-025-02544-x

**Published:** 2025-05-19

**Authors:** Magdalena L. Warren, Kaoru Tsuji, Leslie E. Decker, Manabu Kishi, Jihoon Yang, Adina C. Howe, Tadashi Fukami

**Affiliations:** 1 Department of Biology, Stanford University, Stanford, CA 94305 USA; 2 Center for Ecological Research, Kyoto University, 2-Hirano, Otsu, Shiga 520-2113 Japan; 3 Department of Biology, Graduate School of Science, Kobe University, Kobe, 657-8501 Japan; 4 Laboratory of Japanese Apricot, Wakayama Fruit Tree Experiment Station, 1416-7 Higashi-Honjo, Minabe-cho, Hidaka-gun, Wakayama, 645-0021 Japan; 5 Laboratory of Persimmon and Peach, Wakayama Fruit Tree Experiment Station, 751-1 Oki Aridagawa-cho, Arida-gun, Wakayama, 643-0022 Japan; 6 Department of Agricultural and Biosystems Engineering, Iowa State University, Ames, IA USA; 7 Department of Earth System Science, Stanford University, Stanford, CA 94305 USA

**Keywords:** Microbial ecology, Honeybee microbiome, Nectar microbiome, Plant-pollinator-microbe interactions

## Abstract

Bacteria in the honeybee gut are a well-recognized factor affecting bee health. However, the primary focus of this research has been the hindgut, while the crop, or honey stomach, is assumed to be dominated by environmentally acquired transient taxa that matter little to the bees. To evaluate this assumption, we examined bacterial taxa in the crop and mouth of *Apis mellifera* and *A. cerana japonica* foragers and in the nectar of *Prunus mume* flowers visited by the bees in the Minabe-Tanabe region of Japan. We found that in bacterial composition, the crop was distinct from both the mouth and the nectar, whereas mouth and nectar samples were indistinguishable. Furthermore, the crop remained similar in bacterial composition and diversity, while the mouth showed a sharp drop in alpha diversity and a large increase in beta diversity, from summer to winter. These results refute the conventional assumption, suggesting instead that the crop contains a conserved bacterial community largely distinct from environmental taxa. We also found that strains of a crop-associated species, *Apilactobacillus kunkeei*, could be season- and host species-specific. Together, these findings suggest that crop-associated bacterial communities should be studied further to better understand the relationship between honeybees and their gut bacteria.

**Supplementary Information:**

The online version contains supplementary material available at 10.1007/s00248-025-02544-x.

## Introduction

Microbes in the honeybee gut are receiving increasing attention not only as a factor affecting the health of the agriculturally vital insects, but also as a model system for gaining basic understanding of gut-associated microbiota [1–3]. One component of the honeybee gut is used for the transport of liquids. This organ, which is an expandable portion of the esophagus hereby termed the crop, but also known as the honey stomach [1], contains species such as *Apilactobacillus kunkeei* and *Bombella apis* that may affect the survival and pathogen resistance of the foraging adults that host them and the larvae that the adults feed [1, 4–6]. However, microbes in the crop are not as well studied as those in the hindgut, which are generally considered more important to honeybee health [2, 7, 8].

Past research has found small numbers of bacteria in the crop [9, 10], many of which are also found in the hindgut, hive environment, food stores, and floral nectar, suggesting that the crop may mostly contain environmentally acquired transient microbes [1, 9, 11, 12]. Given its function for temporary storage and transport of floral nectar [9], it seems reasonable to assume that the crop is occupied mainly by the transient microbes acquired from the environment via foraging of nectar [11, 12]. One way to investigate this assumption of environment–crop matching is to compare microbes in the crop of foraging bees and those in their mouth as well as in the floral nectar they forage for. If the assumption is true, the crop should be similar to both the mouth and the floral nectar in bacterial composition [4, 13]. To our knowledge, no study has directly compared bacteria in crop, mouth, and nectar for this purpose, leaving untested the assumption that the crop microbiota is dominated by transients environmentally acquired via foraging.

Here, we test the hypothesis that crop-associated bacteria show species composition that mirror those observed in mouth- and nectar-associated bacteria. To this end, we compare crop-, mouth-, and nectar-associated bacteria in samples that were collected simultaneously at the same locations. Our crop and mouth samples come from actively foraging adults of the introduced *Apis mellifera* and the native *A. cerana japonica* (hereafter *A. cerana*) collected in the Minabe-Tanabe region of Wakayama Prefecture in Japan. In this region, farmers use both species of honeybees to pollinate the winter-blooming Japanese apricot, *Prunus mume* [14]. Our nectar samples come from *P. mume* flowers collected in the winter near the hives of the *A. mellifera* and *A. cerana* bees that we caught for crop and mouth sampling.

## Materials and Methods

### Study Sites

**Fig. 1:**
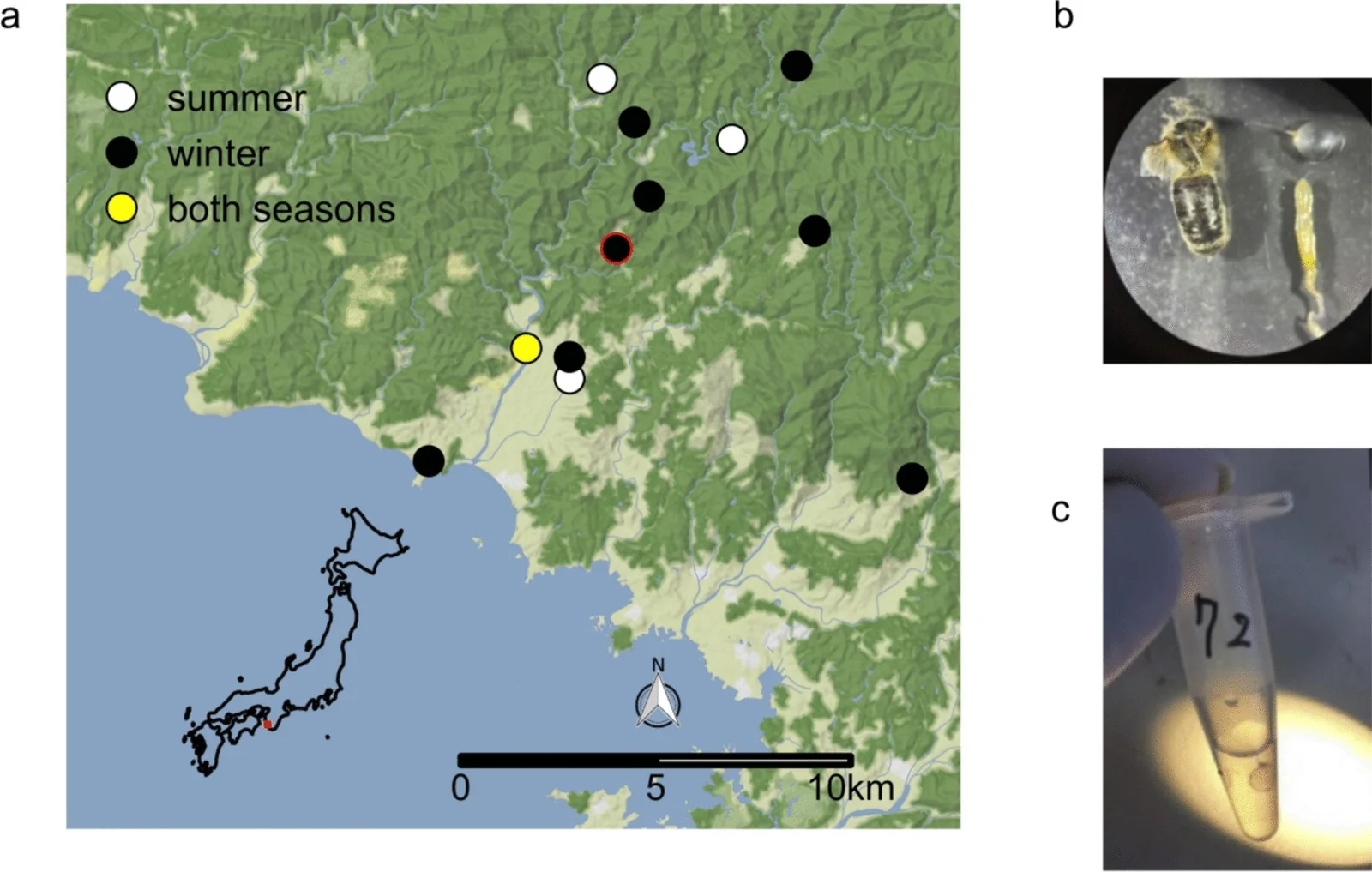
Collection sites and crop dissection. **a** Foraging adults of *Apis cerana* and *A. mellifera* were collected from a total of 12 sites (see Table S1) in or near Japanese apricot orchards in the summer of 2018 and the winter of 2019 in the Minabe-Tanabe region, the location of which is indicated by the red square on the map of Japan. Nectar samples were also collected from apricot flowers at each of the winter collection sites. Sites are labeled with circles colored by collection season (summer = white, winter = black, both seasons = yellow). The location of the Japanese Apricot Laboratory is indicated by the red outline of the Higashi-Honjo site. **b** The crop was distinctly separated from the rest of the alimentary tract. **c** The crop of each honeybee was sterilely dissected and placed in an aliquot of sterile water

Japanese apricot orchards in the Minabe-Tanabe region are embedded within a mountainous countryside landscape that has recently become internationally recognized. In 2015, the Food and Agricultural Organization designated the region as a Globally Important Agricultural Heritage System for the agricultural practice in the region that embraces both human needs and biodiversity conservation. Many of the apricot orchards are located adjacent to natural and managed forests, which are thought to provide stable habitats for the native* A. cerana*. The two *Apis* species are the main pollinators of the apricot, which blooms from mid or late February to early or mid-March, during which few other flower-visiting insects are available [14]. In the winter, *A. mellifera* colonies are supplemented with a sugar solution, while *A. cerana* colonies are not. Although there may have been several different blooming species during our collections, particularly in summer, our interest in nectar focused on the economically important *P. mume,* which was the only primary nectar source available to the bees while *P. mume* was in bloom in the study region*.* All our bee and nectar collection sites were located within this landscape, but at least 1.0 km away from their respective nearest neighboring collection site in each season (Fig. 1a, Table S1).

### Bee Collection

We collected crop and mouth samples not just in winter during the *P. mume* flowering season, but also in summer, to study the influence of seasonal changes on the bacterial composition of the crop and mouth samples. Hives of *A. cerana*, many of which were made of carved tree trunks, were maintained by local beekeepers throughout the year in the region. In contrast, most *A. mellifera* hives in the region were transported elsewhere in the country while *P. mume* was not in bloom. However, some were kept locally throughout the year, allowing us to collect bees in both seasons. One site (Nishi-Honjo) was available for bee collection in both summer and winter, allowing bacterial taxa from the same site and hives to be compared between seasons. In addition, at two of our sites (Higashi-Honjo and Kamihaya), we were able to collect both *A. cerana* and *A. mellifera*, enabling comparison of the two species collected at the same locations (Table S1).

A total of 12 *P. mume* orchards in the study region were visited in the summer of 2018 and the winter of 2019. We collected 44 *A. cerana* and 28 *A. mellifera* in the summer of 2018 and 82 *A. cerana* and 67 *A. mellifera* in the winter of 2019 from hives in or near these apricot orchards (Table S1). *Apis mellifera* were collected from six sites and *A. cerana* were collected from nine sites, but both species were collected from only two sites, Higashi-Honjo and Kamihaya, and only Nishi-Honjo was sampled in both seasons. At each collection site, there were about 4 to 10 *A. cerana* hives or 10 to 40 *A. mellifera* hives. Honeybees were collected as they flew near their hives located within or near the orchards. At orchards where the beekeepers maintained *A. ceran*a hives, *A. cerana* bees were collected near their hives whose locations varied from tens of meters to about 100 m from the orchard and were often situated at the edge of the local forest. The collected bees were individually placed in a sterile, sealed plastic vial immediately after the collection. The vials were kept for up to 2 h in a cool box with ice packs in it for transport to the Japanese Apricot Laboratory located within 100 m from one of the collection sites (Higashi-Honjo) (Fig. 1a).

After we brought the collected bees into the laboratory in the Japanese Apricot Laboratory facility, the bees were kept cool in the box for up to two additional hours so that the bees would stay sluggish for ease of handling. The captured bees were then taken out of the vials, and their wings were fixated with adhesive tape to facilitate handling. The taped bees were made to drink approximately half of 20 µL of sterile 20% sucrose solution placed on the lid of a sterile 1.5-mL microcentrifuge tube. After the feeding, the remaining solution was placed in the microcentrifuge tube with the lid now closed. These solutions were later used to study the bacteria associated with the mouth. We refer to these solutions as mouth samples. As we had the bees extend their proboscis into the sterile sucrose solution, our method is likely to have captured mostly the bacteria from the surface of the proboscis rather than those present within the mandibles.

Immediately after the sampling of mouth-associated bacteria, we dissected the bees by gently pulling the abdomen using forceps to expose the crop, making sure the crop did not touch other parts of the bee body. We then removed the crop from the bee body using another set of forceps (Fig. 1b). Both forceps were sterilized before each use by dipping in 70% ethanol and then flaming. Each crop sample was separately placed in 40 µL of sterile water within sterile microcentrifuge tubes (Fig. 1c) and homogenized using a sterile plastic pestle. All mouth and crop samples were immediately stored at − 80 °C until further processing.

We collected crop and mouth samples not just in winter during the *P. mume* flowering season, but also in summer, to determine whether crop and mouth differed in the way bacterial composition changed from summer to winter. Hives of *A. cerana*, many of which were made of carved tree trunks, were maintained by local beekeepers throughout the year in the region. In contrast, most *A. mellifera* hives in the region were transported elsewhere in the country while *P. mume* was not in bloom. However, some were kept locally throughout the year, allowing us to collect bees in both seasons. One site (Nishi-Honjo) was available for bee collection in both summer and winter, allowing bacterial taxa from the same site and hives to be compared between seasons. In addition, at two of our sites (Higashi-Honjo and Kamihaya), we were able to collect both *A. cerana* and *A. mellifera*, enabling comparison of the two species collected at the same locations (Table S1).

In this study, we did not examine mid- or hindgut samples, since our hypothesis focused on the relationship between the mouth, crop, and nectar. However, investigating the communities in the mid- or hindgut along with those in the crop or mouth would be an interesting direction for future studies.

### Nectar Collection

In addition to the mouth and crop samples, we also collected samples of *P. mume* floral nectar on the same days the bees were collected in the winter of 2019. At each of the nine orchards where we sampled bees that winter (Table S1), we collected 16 nectar samples (except at one site, Kamihaya, where we instead collected 24 nectar samples), for a total of 152 nectar samples. Each sample was collected by probing 10 flowers on a randomly selected branch of an individual *P. mume* tree with a sterile 0.5-µL microcapillary tube and dispensing the collected nectar into a PCR tube containing 40 µL of PCR-quality sterile deionized water. The collected nectar samples were immediately placed in a box with ice packs during the transport to the laboratory. Within 6 h, the nectar samples were brought back to the Japanese Apricot Laboratory. The samples were kept at − 80 °C until further processing for DNA extraction, except during a 1-day transport to the laboratory at Kyoto University in Shiga, Japan, and another 1-day transport from there to Stanford University in California, USA, during which the samples were kept at about − 5 °C.

### Microbial DNA Analysis

Using the nectar and bee samples obtained above, we conducted microbial DNA extraction, bacterial amplicon sequencing, quantitative real-time PCR (qPCR) of 16S rRNA gene, and processing of the amplicon sequencing data (for details, see Text S1).

### Statistical Analysis

All analyses were conducted using R version 4.0.4 [15]. The ASV_97_ (amplicon sequence variants clustered with a 97% similarity threshold; for details, see Text S1) dataset was analyzed with the phyloseq and vegan packages [16, 17]. Venn diagrams of sample types and seasons at the Genus level were created using the MicrobiotaProcess package [18, 19]. Differential abundances of bacteria agglomerated to the Genus level in each sample type were compared within sampling season with the DESeq2 package version 1.30.1 [20]. The random effect of site could not be incorporated into the DESeq design as this is a limitation of the package.

The taxa identified as significantly differentially abundant in the different contrasts (i.e., winter mouth vs. winter crop, winter nectar vs. winter crop, winter mouth vs. winter nectar, and summer crop vs. summer mouth; Fig S1) were combined, and the unique taxa extracted. Of these taxa, those that were present in at least 50% of the sample type groups (Table S2) were further analyzed with a heatmap. The heatmap was created with the ComplexHeatmap package [21]. The differentially abundant taxa in the heatmap were arranged according to their phylogeny, which was inferred using a GTR model in the phangorn package [22]. The samples in the heatmap were clustered based on a weighted UniFrac distance matrix and visualized in a cladogram [23].

Alpha diversity (Shannon index) in each sample was calculated and compared by fitting a linear mixed effects model of Shannon index as the response, season and bee species as fixed effects, and site as a random effect with 2000 parametric bootstraps [24]. Shannon–Weaver evenness was calculated using the evenness function from the microbiome package [25]. Permutation multivariate analysis of variance using a weighted UniFrac distance matrix [23] was used to test the fixed effects of season, sample type, site, and host species on bacterial ASV_97_ composition. The beta diversity of relative abundance and absolute abundance samples was visualized with a weighted UniFrac principal coordinate analysis, and individual sample distances from the centroid and the homogeneity of dispersion of the samples were calculated. We also did multinomial species classification method (CLAM) tests, using the clamtest package [26], to identify ASVs_97_ found more frequently in either winter or summer, those found frequently in both seasons, those too rare to categorize as summer- or winter-associated, and generalists.

### *Apilactobacillus Kunkeei* Strain-Level Phylogenetic Analysis

Fifty microliters of each thawed honeybee crop sample was aliquoted onto De Man, Rogosa and Sharpe (MRS) agar (Sigma-Aldrich), spread thoroughly using a sterile loop, and incubated aerobically at 37 °C for approximately 48 h. Colony PCR was conducted on isolated colonies from these cultured plates to amplify several housekeeping genes. A multi-gene phylogeny of isolates identified as *Apilactobacillus kunkeei* was created with *Apilactobacillus apinorum* Fhon13 (Table S4) [27] as the outgroup (for details, see Text S2).

## Results

### Crop–Mouth–Nectar Comparisons

**Fig. 2:**
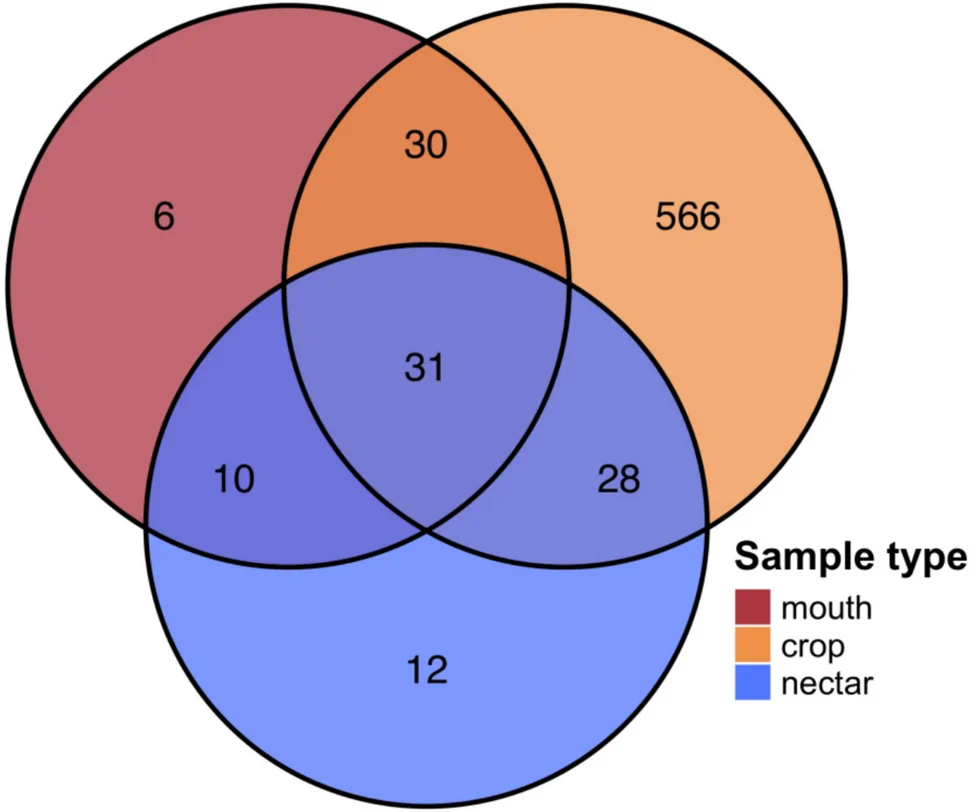
The crop contains more common and constant ASVs_97_ than the mouth and the nectar. Only 9% of 655 winter crop ASVs_97_ were shared with the mouth or nectar samples. In contrast, the mouth and nectar shared about 50% of their ASVs_97_ (77 and 81 ASVs_97_, respectively). Venn diagrams are colored by sample type with ASV_97_ counts in bold

**Fig. 3:**
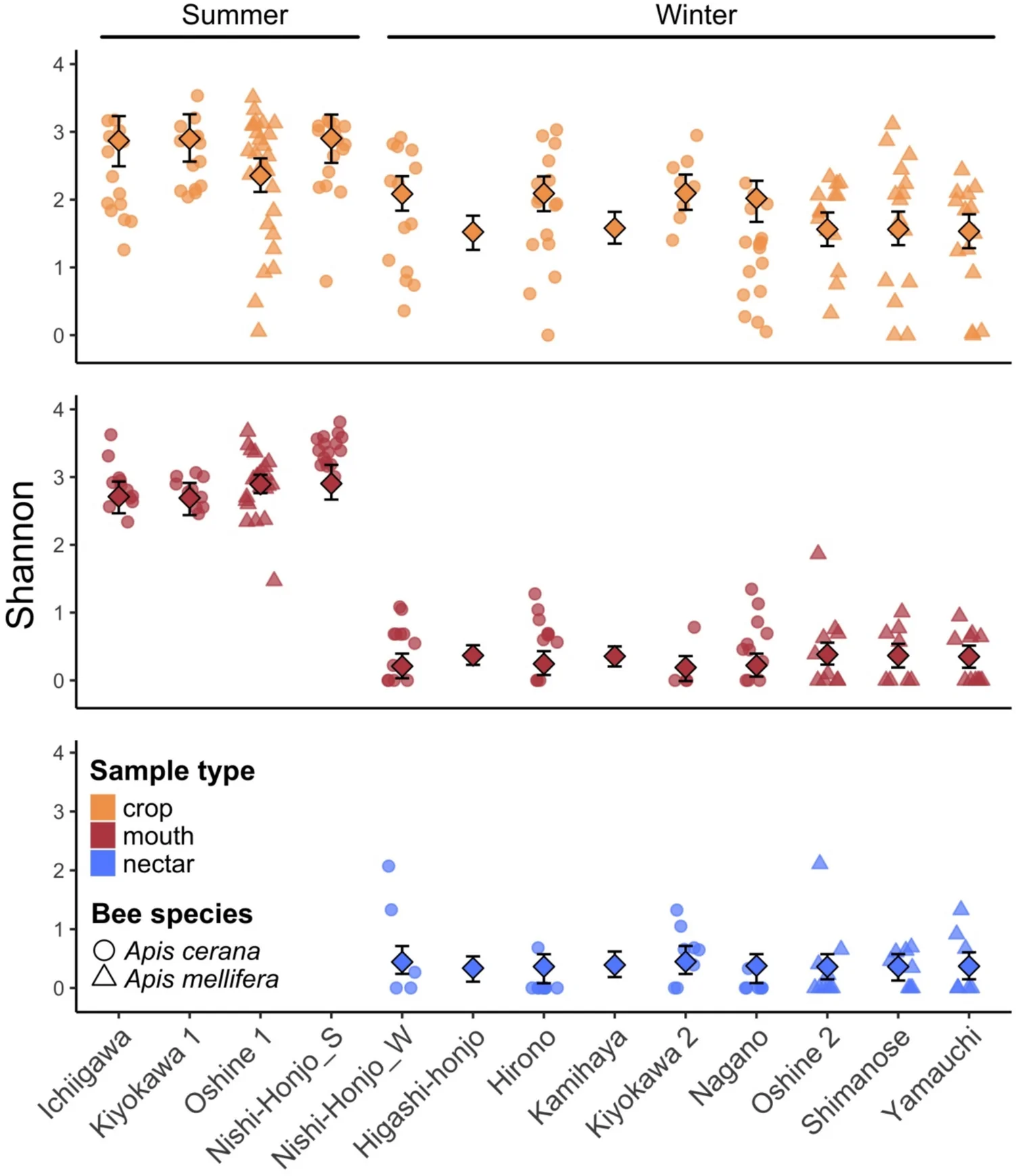
The crop presents more consistent species richness. Species richness (Shannon index) in the crop varied less from summer to winter than the mouth, and winter mouth and nectar samples had similar species richness across sites. Nishi-Honjo_W and Nishi-Honjo_S are the samples collected at the Nishi-Honjo site (see Table S1) in winter and summer, respectively. Each data point represents a single sample collected from the labeled site, colored by sample type, and shaped by host species. Mean diversity is depicted by the large center point, and the error bars depict the bootstrapped 95% confidence interval for samples from each site

Among winter samples, we found more than seven times as many bacterial ASVs_97_ in the crop (a total of 655) as in the mouth and the nectar (a total of 77 and 81, respectively) (Fig. 2). Mean ASV_97_ diversity per sample was about six times higher in the crop (1.78) than in the mouth (0.37) and the nectar (0.36) (crop*–*mouth: Welch’s *t* = 17.1, *p*-value < 0.001; crop*–*nectar: Welch’s *t* = 15.8, *p*-value < 0.001). Similarly, mean evenness was about two times higher in the crop (0.74) than in the mouth (0.42) and the nectar (0.38) (crop*–*mouth: Welch’s *t* = 6.8, *p*-value < 0.001; crop*–*nectar: Welch’s *t* = 7, *p*-value < 0.001) (Fig. 3).

**Fig. 4:**
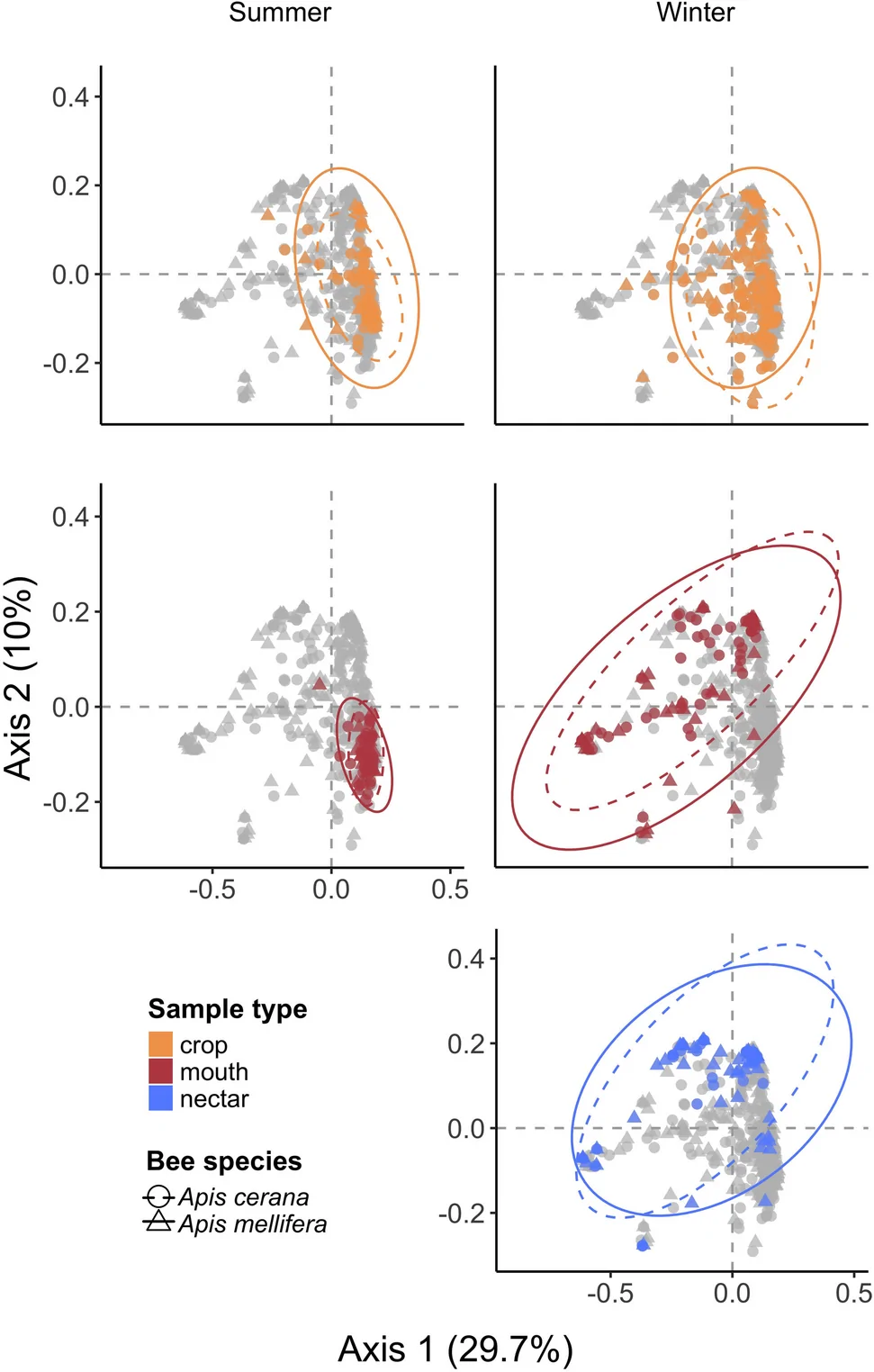
Principal coordinate analysis of all samples presents similar composition across season in the crop but not the mouth. When the composition of all samples from both seasons were compared, winter mouth and nectar samples were highly similar (PERMANOVA sample type: *R*^2^ = 0.07, *p*-value = 0.001, season: *R*^2^ = 0.05 *p*-value = 0.001, host species: *R*^2^ = 0.003, *p*-value = 0.154). Each dot represents a sample from an individual bee, colored by sample type. *Apis mellifera* samples are depicted with triangles and the solid ellipse whereas *Apis cerana* samples are depicted with circles and the dashed ellipse. Ellipses mark the 95% confidence interval, and axes are labeled with corresponding percent variation explained

Of sample type (crop, mouth, or nectar), bee species (*A. mellifera* or *A. cerana*), and sampling site (Fig. 1a), only sample type was a significant predictor of bacterial ASV_97_ composition (PERMANOVA *R*^2^ = 0.10, *p*-value = 0.001). Specifically, mouth and nectar samples were more similar to each other in bacterial composition than either mouth and crop samples or nectar and crop samples were to each other (Fig. 4; PERMANOVA mouth*–*nectar *R*^2^ = 0.03, *p*-value = 0.002; mouth*–*crop *R*^2^ = 0.11, *p*-value = 0.001; nectar*–*crop *R*^2^ = 0.08, *p*-value = 0.001).

**Fig. 5:**
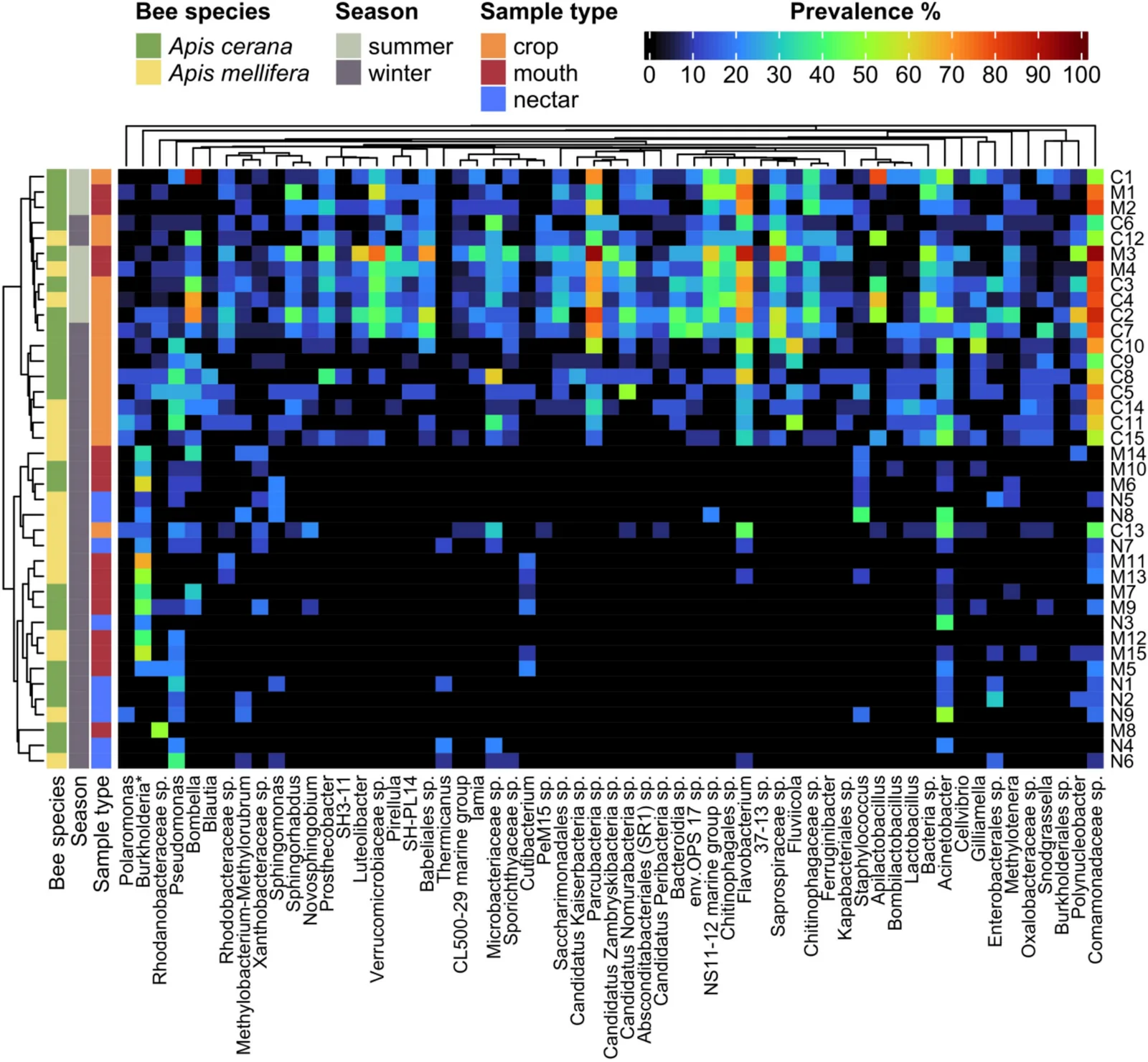
There is a greater prevalence of differentially abundant taxa among crop groups than mouth and nectar groups. Samples were grouped by season, sampling site, host *Apis* species, and sample type as illustrated by colored bars to the left of the heatmap and described in Table S2. For ease of visualization, heatmap columns are differentially abundant taxa that were present in at least 50% of one of the sample groups, e.g., at least 50% of all the mouth groups. Burkholderia* denotes the genus Burkholderia-Caballeronia-Paraburkholderia. The full heatmap with individual samples and all differentially abundant taxa is found in Figure S3. The ASVs_97_ were agglomerated to genus, colored by prevalence within groups, and arranged according to their phylogeny. The y-axis dendrogram depicts the weighted UniFrac distance between samples

In fact, most ASVs_97_ in the crop were distinct from those in the mouth and the nectar, with only 9% (4–8% in *A. cerana* and 6–9% in *A. mellifera*; Fig. S2) of the crop ASVs_97_ also present in the mouth or the nectar (Fig. 2). Taxa frequently found in the crop, such as *Bombella*, *Gilliamella*, *Lactobacillus*, and *Snodgrassella*, were mostly absent in the nectar and only infrequently present in the mouth (Figs. 5 and S3). Furthermore, some ASVs_97_ belonging to *Acinetobacter,* Comamonadaceae, and *Flavobacterium* were consistently found in the crop at all local sites examined, whereas *Acinetobacter* was present in only 7 of the 9 nectar sites and 11 of the 15 mouth sites, and there were no other taxa that were present in mouth or nectar samples at all sites.

These results held true even when we excluded those taxa that are known as the core phylotypes of the hindgut bacterial community (*Gilliamella*, *Lactobacillus*, and *Snodgrassella*) from the dataset used for the analysis (Fig. S4).

### Summer–Winter Difference in Crop vs. in Mouth

**Fig. 6:**
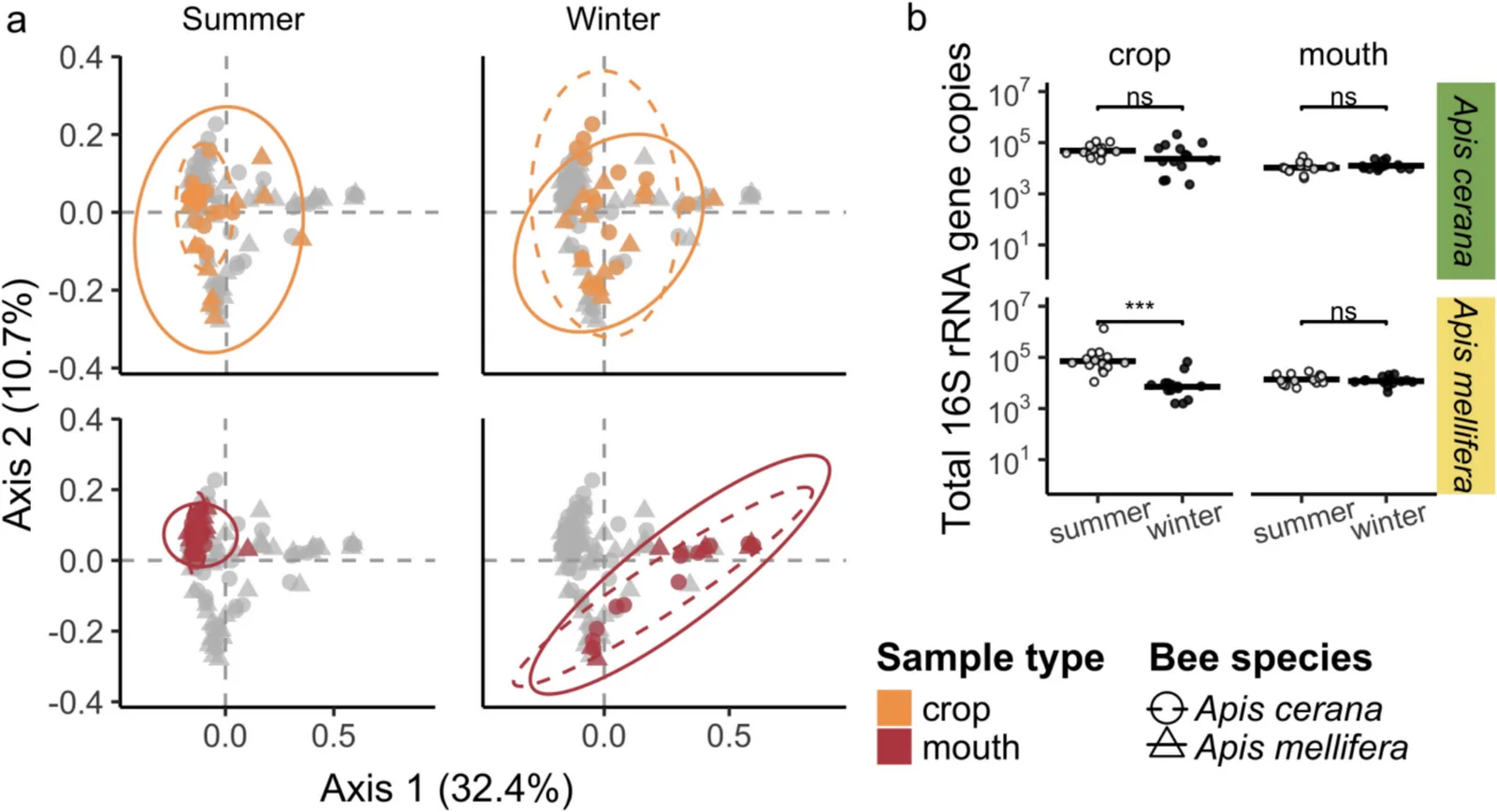
Consideration of absolute abundance estimates in analysis of bacterial composition. **a** As in Fig. 4, principal coordinate analysis of samples that takes into account absolute total bacterial abundance indicates greater compositional similarity between crop samples than mouth samples across season. Specifically, mouth samples cluster more closely in the summer and are more dispersed in the winter than the crop samples (betadisper distance to median: summer-mouth = 0.21, winter-mouth = 0.40, summer-crop = 0.26, winter-crop = 0.32; betadisper Tukey HSD summer–winter mouth: *p*-value < 0.001, summer–winter crop: *p*-value = 0.008). Overall, sample type, season, and site but not host species are significant predictors of composition (PERMANOVA sample type: *R*^2^ = 0.03, *p*-value = 0.008, season: *R*^2^ = 0.12, *p*-value = 0.001, host species: *R*^2^ = 0.01, *p*-value = 0.191, site: *R*^*2*^ = 0.09, *p*-value = 0.004). Each point represents a single sample, is color coded by sample type, and shaped by bee species. Ellipses represent the 95% confidence interval for each bee species. Percent variation explained by each axis is labeled. **b** Mouth samples tended to have a more even, lower bacterial load across season than did crop samples. The *Apis mellifera* but not the *Apis cerana* crop samples had a higher bacterial load in the summer than the winter. The mouth maintained the same bacterial load across seasons. Total 16S rRNA gene copies per 10 µL are presented on the log scale, with each point depicting a sample from an individual bee colored by season (summer = white, winter = black). Wilcoxon test significance is labeled with *** = *p*-value < 0.001 and ns = not significant

Although ASV_97_ richness was higher in summer than in winter in both the crop and the mouth, the magnitude of this difference was eight times greater in the mouth than in the crop (Figs. 3 and S5). In terms of ASV_97_ composition of the crop and mouth samples, most of the crop samples were clustered together regardless of season in both the relative and absolute abundance datasets (relative abundance: winter vs. summer, PERMANOVA *R*^2^ = 0.03, *p*-value = 0.001; absolute abundance: winter vs. summer, PERMANOVA *R*^2^ = 0.04, *p*-value = 0.021), whereas the mouth samples formed two distinct clusters, corresponding to summer and winter (relative abundance: PERMANOVA *R*^2^ = 0.20, *p*-value = 0.001; absolute abundance: PERMANOVA *R*^2^ = 0.31, *p*-value = 0.001; Figs. 4, 5, and 6a). These two mouth clusters differed in the amount of bacterial composition variation among samples, with winter mouth samples showing higher variation than summer mouth samples (Figs. 4 and 6a) despite no significant difference between them in total bacterial load (Fig. 6b). In contrast to the mouth, the bacterial load in the crop was significantly higher in the summer than in the winter in the *A. mellifera* crop (but not in *A. cerana* crop; Fig. 6b).

**Fig. 7:**
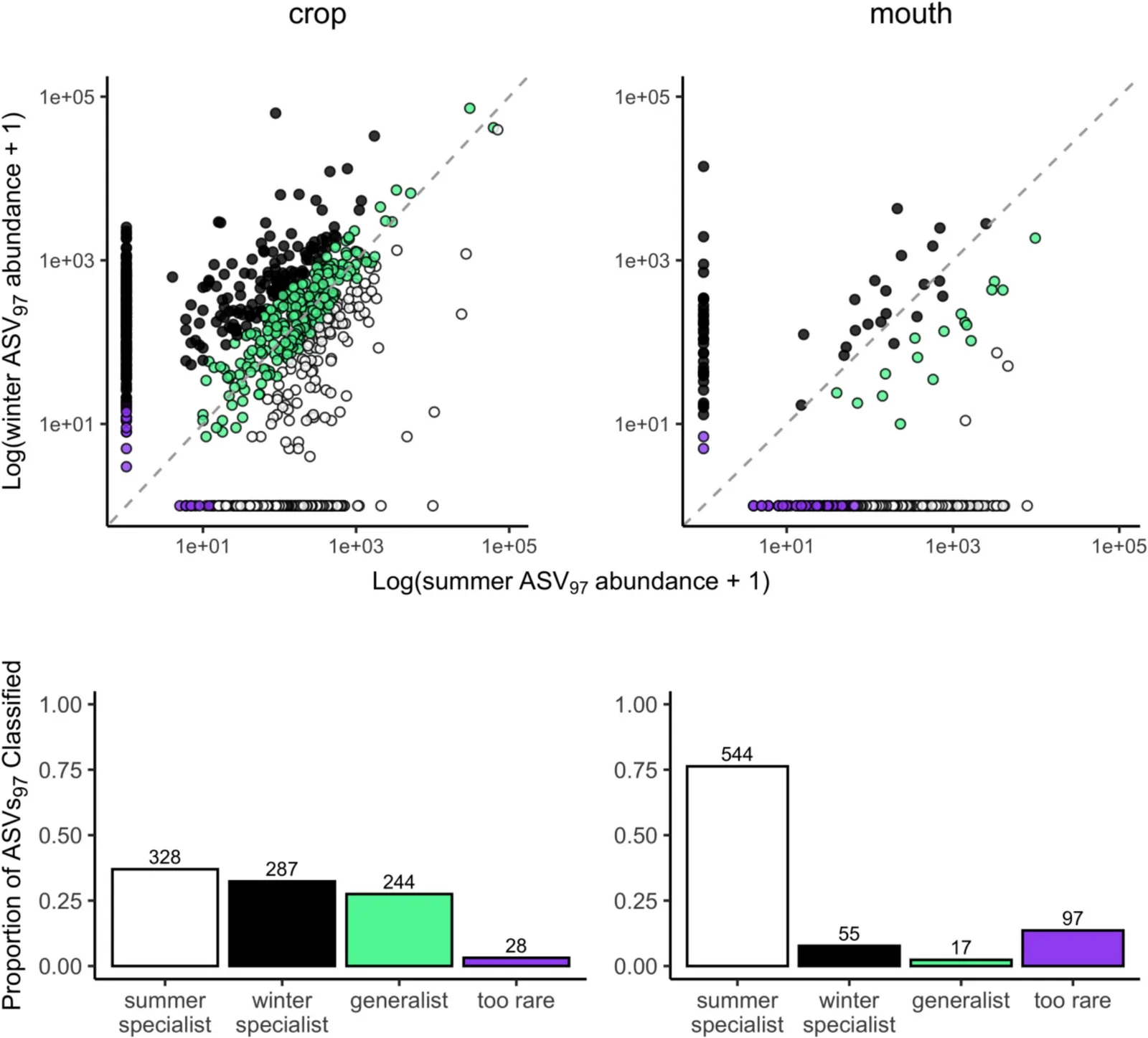
There are bacteria that have high affinity for the crop. Generalist bacteria, i.e., those with no special affinity to one environment or, in this case, season, are greater between the summer and winter crop samples (about 27% of the ASVs_97_) than the corresponding mouth samples. The mouth presents only about 2% generalists, with most ASVs_97_ classified as summer-associated (76%). Scatterplots depict the log abundance of each ASV_97_, with each point representing a single ASV_97_ color coded by its category. ASVs_97_ from both *A. mellifera* and *A. cerana* are included. The identity line is plotted in a dotted grey pattern. The proportion of ASVs_97_ classified in each category is depicted in the corresponding bar plots below the scatterplots, with categories colored as in the scatterplot

The CLAM test [26] identified 28% of the ASVs_97_ in the crop as generalists, meaning that they were similarly frequent in winter and summer. In contrast, the CLAM test classified only 2% of the ASVs_97_ in the mouth as generalists, with most others identified as either summer- or winter-associated (76 and 8%, respectively; Fig. 7).

### *Apilactobacillus kunkeei* Strains in the Crop

**Fig. 8:**
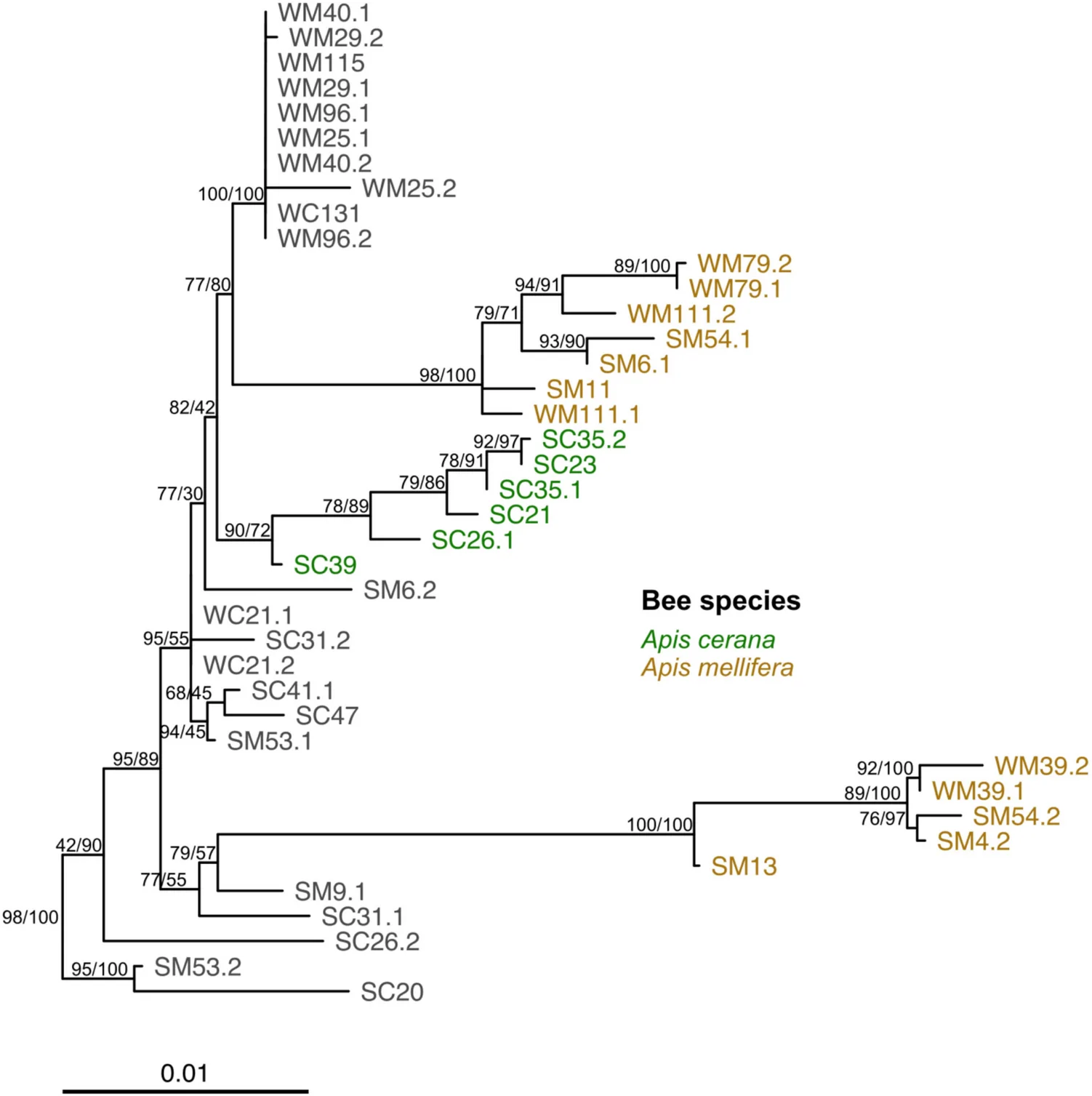
Multi-gene maximum likelihood tree of *Apilactobacillus kunkeei* isolates contains *Apis* species specific clades. The phylogeny is the result of a multi-gene alignment partitioned analysis of 49 *A. kunkeei* isolates and *A. apinorum* Fhon13 (see Tables S3 and S4). In the depicted tree, substitution frequencies are represented by branch length. Isolates are labeled starting with an “S” for summer or “W” for winter, followed by a “C” for *Apis cerana* and an “M” for *Apis mellifera* host species, the host ID number, and lastly the isolate number. Isolates that occurred in both species are colored in grey whereas those that are phylogenetically similar with high statistical support and associated with a single host species are colored by host species. Statistical support was estimated from 1000 Shimodaira-Hasegawa-approximate likelihood ratio test (SH-aLRT) pseudo replicates and ultrafast bootstraps (UFboot). Nodes with an SH-aLRT score greater than 0 are labeled with respective SH-aLRT/UFboot values. *Apilactobacillus apinorum* root is not depicted for ease of visualization. Figure S6 presents the full tree with statistical support on all nodes and *A. apinorum* branch

The multi-gene tree of *Apilactobacillus kunkeei* strains suggested potential season- and *Apis* species-specificity. Specifically, the tree had one clade consisting of only isolates from summer *A. cerana* samples and two other clades consisting of isolates from summer and winter *A. mellifera* samples (Figs. 8 and S6). Those associated with *A. cerana* originated from five bees, three from Nishi-Honjo and two from Ichiigawa. Those associated with *A. mellifera* originated from eight bees: five summer bees from Oshine 1 and three winter bees, one each from Shimanose, Yamauchi, and Kamihaya.

## Discussion

Based on the conventional assumption that bacterial assemblage in the crop primarily consists of transient microbes acquired via nectar foraging [3, 9, 27, 28], we expected a high degree of bacterial overlap among nectar, mouth, and crop samples. We did find high overlap between the nectar and the mouth in the winter, but to our surprise, most of the crop taxa were clearly distinct from those found in the nectar and the mouth (Figs. 2 and 5) [11]. Several ASVs_97_ of *Acinetobacter* and *Pseudomonas* that were relatively prevalent in both crop and mouth samples in our study have been commonly found in nectar as well [29–31]. However, most of the taxa that were more prevalent in the crop were rare or entirely absent in nectar and mouth samples (Figs. 5, S2, and S3) [11].

In addition, our results indicate that the crop maintained a high degree of taxonomic consistency between winter and summer despite differences in bacterial load in *A. mellifera* crop samples, a pattern that is in sharp contrast to the large compositional changes seen in the mouth between seasons and between bee species (Figs. 3, 4, 6a, and 7). Taxa common in the crop in both seasons included Comamonadaceae, *Acinetobacter*, Saprospiraceae, *Flavobacterium*, *Gilliamella*, *Bombella*, *Apilactobacillus*, *Pseudomonas*, *Fluviicola*, *Polynucleobacter*, *Snodgrassella*, and Microbacteriaceae species (Fig. 5). *Apilactobacillus* and *Bombella*, previously called *Parasaccharibacter apium* [5, 32, 33], which are among the most commonly reported taxa in the crop of foraging adults and have been suggested as beneficial to host health, have also been found frequently in food stores, larvae, and queens [3–5, 9, 27, 34–36]. We recently found that crop bacterial communities were more variable than those of the gut, but that they were more consistent across collection sites than fungal communities [12]. These results support the findings that bacterial composition in the crop is less deterministic than that of the intestines, but more deterministic than that in the mouth or nectar. Although the data we present in this study are correlational, preventing us from establishing causal relationships, we speculate that the crop may serve as a consistent reservoir of these taxa that can influence the health of the adult bees and the larvae that they tend [3, 27].

We also found evidence for potential strain-level specificity among *Apilactobacillus kunkeei* isolates from collected foragers. One clade was made up of summer *A. cerana* isolates from two different sites, and two others contained only *A. mellifera* isolates from both seasons (Figs. 8 and S6). Although a larger sample size than we had in this study as well as full genome sequencing are needed to ascertain the putative strain-level differences, previous studies have shown differences in specific house-keeping genes of core gut microbes to suggest strain-level host specificity between *Apis* and *Bombus*, and even among *Apis* species [37, 38]. Our isolates seemed to present similar patterns to those described in these previous studies, with some of our isolates clustering more frequently by host species than by site, potentially suggesting host specificity and possibly specific host-symbiont relationships between *Apis* species and *A. kunkeei* strains. However, this possibility remains speculative at this stage. It is possible that several of these isolates were obtained via environmental acquisition.

Several caveats should be considered in interpreting our findings. First, our analysis is mostly based on relative abundance [39], even though we used qPCR to attempt to estimate at least total absolute abundance in crop and mouth samples (Fig. 6b). The bacterial load in our crop samples was in line with estimates previously reported, i.e., lower than the load reported in hindgut studies [10], and we did see similar bacterial composition patterns in our analysis of both relative and absolute abundance (Figs. 4 and 6a), but absolute abundance [13, 39] can differ even when relative abundance is the same. Second, we found that the crop did not consistently have a higher bacterial load than the mouth (Fig. 6b). Although resident populations can be persistent regardless of size, population size of each taxon might fluctuate more across seasons than is suggested by the relative abundance. Third, we had only one site, Nishi-Honjo, where bees were sampled in both summer and winter. A greater and more even number of bees at each site in multi-year seasonal sampling would have made our results most robust. Nevertheless, our analysis of several hundred bee and nectar samples all sampled within the same landscape calls for further investigation into the hypothesis that crop communities simply reflect what is available in the environment. Lastly, we did not sample the hive, where the bees could have acquired environmental bacteria [9]. However, the close compositional match between mouth and nectar samples (Fig. 4) indicates that the nectar samples captured much of the taxa found in the mouth samples.

In conclusion, our results suggest that the composition of crop bacteria may be more deterministic than generally assumed, questioning the notion that crop bacteria are environmentally acquired transients that are unimportant to the bees. Although less well studied than midgut and hindgut microbes, a small but increasing number of recent studies indicate that some crop microbes do alter the health of bee individuals [6, 40–42]. Our data reinforce the idea that it would help to study how microbial communities are assembled and maintained not just in the midgut and the hindgut, but also the crop, to better understand the relationship between honeybees and their gut microbes.

## Supplementary Information

Below is the link to the electronic supplementary material.

Supplementary file1 (DOCX 41072 KB)

## Data Availability

The datasets generated during and/or analyzed during the current study are available in GenBank, https://www.ncbi.nlm.nih.gov (BioProject ID no. PRJNA1076572 and accession no. PP412473-PP412519, PP341993-PP342038, PP382363-PP382409, PP382266-PP382314, PP382315-PP382362), or are included in this published article and its supplementary information files. All custom scripts are available on GitHub at https://github.com/mlwarren20/ume_bee_analysis.
