# Supplementary material for: Bacteria in Honeybee Crops Are Decoupled from Those in Floral Nectar and Bee Mouths
Source: Microb Ecol. 2025 May 19;88(1):46. doi: 10.1007/s00248-025-02544-x (PMC12089155; doi:10.1007/s00248-025-02544-x)
Supplement: Supplementary file 1 — Supplementary file1 (DOCX 41072 KB) [file 248_2025_2544_MOESM1_ESM.docx]

**Bacteria in honeybee crops are decoupled from those in floral nectar and bee mouths**

**Supplemental Material**

Magdalena L. Warren^1^*^†^, Kaoru Tsuji^1,2,3^*, Leslie E. Decker^1^, Manabu Kishi^4,5^, Jihoon Yang^6^, Adina C. Howe^6^, Tadashi Fukami^1,7†^

^1^Department of Biology, Stanford University, Stanford, CA 94305, USA

^2^Center for Ecological Research, Kyoto University, 2-Hirano, Otsu, Shiga, 520-2113, Japan

^3^Department of Biology, Graduate School of Science, Kobe University, Kobe, 657-8501, Japan

^4^Japanese Apricot Laboratory, Wakayama Fruit Tree Experiment Station, Minabe, Higashi-Honjo 1416-7, Wakayama, 645-0021, Japan

^5^Hidaka Promotion Bureau, Wakayama Prefecture, Gobo, Yukawa 651, Wakayama, 644-0011, Japan

^6^Department of Agricultural and Biosystems Engineering, Iowa State University, Ames, Iowa, USA

^7^Department of Earth System Science, Stanford University, Stanford, CA 94305, USA

* These authors contributed equally to this work

^†^Co-corresponding authors:

[mlwarren@stanford.edu](mailto:mlwarren@stanford.edu); ORCID: 0000-0003-0115-5066

[fukamit@stanford.edu](mailto:fukamit@stanford.edu); ORCID: 0000-0001-5654-478

**Summary**

***Texts***

- **Text S1.** Microbial DNA analysis
- **Text S2.** *Apilactobacillus kunkeei* strain-level phylogenetic analysis

***Figures***

- **Figure S1.** Pairwise sample differential abundance based on DESeq2 analysis. The four panels show comparisons of (**a**) winter crop and winter mouth, (**b**) winter nectar and winter mouth, (**c**) winter nectar and winter crop, and (**d**) summer crop and summer mouth. Only statistically significant differences are plotted and colored by Phylum. Taxa are agglomerated to and labeled by Genus.
- **Figure S2.** Samples collected from *Apis cerana* and *Apis mellifera* sites show the same overall pattern of more common and constant ASVs_97_ in the crop than the mouth and the nectar. (**a**) At *Apis cerana* sites, only 8% and 4% of the winter crop ASVs_97_ were shared with the mouth and nectar samples, respectively, while the mouth and nectar shared about 30% of their ASVs_97_. (**b**) Similarly, at *Apis mellifera* sites, 6 and 9% of the crop ASVs_97_ were shared with the mouth and nectar samples, respectively. Half of the mouth ASVs_97_ were also present in the nectar, while only about 30% of the nectar ASVs_97_ were found in the mouth. Venn diagrams are colored by sample type with ASVs_97_ counts in bold as in Figure 2.
- **Figure S3.** Heatmap as in Fig. 6 except that samples are ungrouped and the heatmap represents presence (in green) and absence (in black) of the taxa in each sample. Also, all differentially abundant taxa (see Figure S1), agglomerated to genus, are depicted.
- **Figure S4.** Exclusion of genera commonly found in the hindgut, specifically *Gilliamella, Snodgrassella,* and *Lactobacillus*, does not change the (**a**) alpha or (**b**) beta diversity trends of the bacterial communities. The crop bacterial alpha diversity and composition remain more constant across season than those of the mouth, while the mouth and nectar present very similar alpha and beta diversity in the winter.
- **Figure S5.** Median coefficients with error bars representing the bootstrapped 95% confidence interval of linear mixed effects model of Shannon diversity with the random effect of site.
- **Figure S6.** (**a**) Cladogram of phylogeny as in Fig. 8 including the outgroup, *Apilactobacillus apinorum* Fhon13, and statistical support from 1000 SH-aLRT pseudo replicates (%), a Bayesian-like transformation of aLRT (aBayes), and 1000 ultrafast bootstraps (%) in that order. (**b**) Multi-gene phylogeny including Fhon13. Branch length represents substitution frequency. Nodes are colored by collection site; sample labels start with an “S” for summer or a “W” for winter samples followed by the host bee ID and the isolate number and are colored by host species.
- **Figure S7.** Melting curves from the qPCR assays.
- **Figure S8.** ASV accumulation curves for (**a**) crop, (**b**) mouth, and (**c**) nectar samples. The colors represent the different samples, and the lines are coded to show the sample-size-based rarefaction or extrapolation sampling curves.

***Data***

- **Data S1**. Data table of apricot orchard collection year, season, latitude, and longitude.
- **Data S2**. Data table with quantitative PCR values and sample type for each sample where the 16S rRNA gene was quantified.
- **Data S3**. Data table with the name of each *Apilactobacillus kunkeei* isolate used in the phylogeny (see Fig. 8 and S6), along with the species and site it was isolated from, and the tag used in the phylogenetic tree.

***Tables***

- **Table S1**. Summary of the bees used in this study.
- **Table S2**. Sample type group IDs and their corresponding attributes.
- **Table S3**. Partition models chosen by IQ-TREE ModelFinder.
- **Table S4**. Genbank accession numbers for all strains used in *Apilactobacillus kunkeei* phylogeny (see Figures 8 and S6). Where a high-quality sequence for a gene could not be obtained the gene’s accession number is labeled with "NA".
- **Table S5**. Quantitative PCR (qPCR) Standard curve information.
- **Table S6.** Summary of DADA2 processing read counts for each sample.

**Text S1**. Microbial DNA analysis

*Microbial DNA extraction*

In our laboratory at Stanford University, the Qiagen DNeasy Blood and Tissue kit and Qiagen Genomic DNA Buffer set (Qiagen, Germantown, MD, USA) were used to extract DNA from all the samples. The extraction followed the gram-positive bacteria protocol along with the following modifications. For each of the crop, mouth, and nectar samples collected, 10 µL of the thawed sample was combined with 2 µL of zymolyase enzyme (MP Biomedicals, Burlingame, CA, USA) within a microcentrifuge tube. Two DNA extraction negative controls, consisting of only the 2 µL of zymolyase enzyme, were included for every 90 samples for a total of 13 DNA extraction negative controls. These mixtures were incubated at 30°C for 30 minutes. Once the incubation period was complete, 10 µL of a 500:1 G2 (from the Qiagen Genomic DNA Buffer set) buffer to RNAse A (Qiagen) mixture was added to each microcentrifuge tube. 20 µL of ProteinaseK (Qiagen) and 180 µL of Buffer ATL (Qiagen) were added to each sample and vortexed to mix. The samples were then incubated at 56°C overnight. The next morning the samples were mixed and centrifuged to collect any condensation on the caps, and 410 µL of AL-Ethanol (Qiagen) mixture were added to each sample and shaken vigorously to mix. The rest of the protocol was followed as stated in the DNeasy Blood & Tissue Handbook (2018) with the only exception being that the final elution of DNA was done with 50 µL of Buffer AE (Qiagen) rather than 200 µL to increase the final DNA concentration in the eluate. The final extracted DNA from each sample was stored in sterile microcentrifuge tubes at 4°C until library preparation.

*Bacterial amplicon sequencing*

The highly variable (V4) region of the 16S rRNA gene was amplified using primers 515F (Parada) (5´-GTGYCAGCMGCCGCGGTAA-3´) and 806R (Apprill) (5´-GGACTACNVGGGTWTCTAAT-3´) [1-3]. Both primers included an Illumina (San Diego, CA, USA) adapter on the 5´ ends used to attach unique Nextera XT indexes for sample identification. Each 10 µL first step PCR amplification consisted of 3.2 µL of PCR-grade water, 5 µL of MyTaq HS Red Mix (Bioline, Taunton, MA, USA), 0.4 µL each of the 10 µM forward and reverse primers, and 1 µL of the extracted DNA sample. Two negative control samples, where the reaction did not include any input DNA, per every 90 experimental samples, were included for a total of 13 first step PCR negative controls. PCR conditions were 95ºC for 2 minutes, followed by 35 cycles of 95ºC for 20 seconds, 50ºC for 20 seconds, 72ºC for 50 seconds, and a final extension at 72ºC for 10 minutes with a final hold at 4ºC. Amplification was confirmed by running samples on an agarose gel, and the resulting amplicons were purified using Sera-Mag SpeedBeads (Sigma-Aldrich, St. Louis, MO, USA). The second step 10 µL PCR amplification consisted of 3.2 µL of PCR-grade water, 5 µL of MyTaq HS Red Mix (Bioline), 0.4 µL each of the combined Nextera XT index primers 1 and 2, and 1 µL of the first step PCR product. Two negative control samples, where the reaction did not include any of the first step PCR product, per every 90 experimental samples, were included for a total of 13 second step PCR negative controls. PCR conditions for this second step were 95ºC for 2 minutes, followed by 8 cycles of 95ºC for 20 seconds, 50ºC for 20 seconds, 72ºC for 50 seconds, and a final extension at 72ºC for 10 minutes with a final hold at 4ºC. Amplification was confirmed by running samples on an agarose gel, the barcoded amplicons were purified once again using Sera-mag SpeedBeads (Sigma-Aldrich), and the resulting clean product was pooled evenly across samples. The pooled samples were combined to a concentration of 4 nM using a Qubit 4 Fluorometer and High Sensitivity dsDNA Assay kit (Thermo Fisher, Waltham, MA, USA), and then the entire amplicon libraries were pooled together. The final DNA concentration and quality of the pooled amplicon libraries were quantified with a Fragment Analyzer (Agilent, Santa Clara, CA, USA), and then sequenced on a MiSeq (Illumina) using a 2x300 cycle sequencing kit with a 15% PhiX spike-in at the Stanford Genomic Sequencing Service Center.

*Quantitative real-time PCR (qPCR) of 16S rRNA gene*

A subset of the crop and mouth samples was selected for 16S rRNA gene quantification. These 112 samples were made up of mouth and crop samples from 28 *Apis mellifera* and 28 *A. cerana* bees, 14 each from the summer and winter sites. To ensure we captured differences in bacterial load in Nishi-Honjo, the one site where we did the crop and mouth sampling in both seasons, we included all samples from this site in this analysis. The remaining *A. cerana* and *A. mellifera* samples were randomly selected from the other sites. The total number of bacterial 16S rRNA genes was quantified in the extracted DNA samples. Targeted genes were amplified using the CFX96^TM^ Real-Time System (C1000TM Thermal Cycler, Bio-Rad, Hercules, CA, USA). All reactions were performed using SsoFast™ EvaGreen® Supermix (Bio-Rad) according to the manufacturer’s instructions and in triplicate using 10 μL of SsoFast EvaGreen Supermix, 0.6 μL of each 300 nM primer, 2 μL DNA samples, and nuclease-free water to a final volume of 20 μL. 16S rRNA genes were amplified using the primer sets 1055YF(5’-ATGGYTGTCGTCAGCT-3’) and 1392R (5’-ACGGGCGGTGTGTAC-3’), with the PCR conditions as follows: 2 minutes at 50°C and 10 minutes at 95°C, followed by 40 cycles of 15 seconds at 95°C and 1 minute at 58°C [4]. The detection limit (1.6 x 10^2^ gene copies per reaction) for quantitative real-time PCR was determined using 55 standard curves for 16S rRNA genes that were constructed using the serial 10-fold dilutions from 10^-1^ to 10^-7^ of known concentrations of synthesized oligonucleotides (Integrated DNA Technologies, Inc., Coralville, IA, USA). The gene copy number was determined from CT values equal to or higher than the negative controls were considered as below the detection limit.

*16S rRNA gene amplicon sequencing data processing*

The sequencing resulted in a mean of 38,702 reads for the crop samples, 19,230 reads for the mouth samples, and 15,352 reads for the nectar samples. The raw amplicon sequences were trimmed using the Cutadapt software [5]. The Divisive Amplicon Denoising Algorithm 2 (DADA2) pipeline [6] was used to merge paired-end sequences, quality filter these sequences, remove chimeric reads, and cluster sequences into amplicon sequence variants (ASVs). The mean number of reads was 32,911 for the crop samples, 17,414 for the mouth samples, and 14,581 for the nectar samples after processing with DADA2. Taxonomy assignments for each ASV were assigned using the SILVA version 138.1 database [7]. ASVs present in the DNA extraction and first and second step PCR negative controls as well as those identified as chloroplast or mitochondria were removed from all samples in the dataset. Any phyla less than 1% abundant among all samples were excluded [8]. This preprocessing resulted in several samples containing no sequence reads. Consequently, these samples were excluded from further analysis, resulting in a total of 218 crop samples, 161 mouth samples, and 82 nectar samples used for downstream analysis. Sampling effort was assessed using ASV accumulation curves created with the iNEXT R package [9]. To minimize grouping ASVs from the same genome into separate clusters [10], ASVs were grouped into ASVs_97_ sequences, as previously described [11], with a 97% similarity threshold using the DECIPHER and speedyseq R packages [12, 13]. Counts for all samples were transformed to relative abundance by dividing the number of reads assigned to each ASV_97_ in each individual sample by the total number of reads in the sample. To convert the counts to absolute abundance, for the subset of samples with qPCR data, we multiplied the total number of 16s rRNA gene copies in each sample by the proportion of each ASV_97_ in said sample.

**Text S2.** *Apilactobacillus kunkeei* strain-level phylogenetic analysis

Half of each single white colony grown on MRS agar was picked and placed in 10 µL of MilliQ (EMD Millipore, Burlington, MA, USA) water, and the other half was streaked onto a new MRS agar plate with a sterile loop. The 16S rRNA gene of these diluted colonies was amplified using the universal primers 27F (5’-AGAGTTTGATCCTGGCTCAG-3’) and 1492R (5’-ACGGCTACCTTGTTACGACTT -3’) in a 25 µL PCR amplification that included 12.5 µL of MyTaq Red Mix (Bioline), 8.75 µL of PCR-grade water, 1.25 µL each of the 10 µM forward and reverse primers, 0.25 µL of dimethyl sulfoxide (DMSO), and 1 µL of each diluted colony. PCR conditions were one 95°C denaturation cycle for 3 min, followed by 35 cycles (30 s at 95°C, 30 s at 50°C, 45 s at 72°C), and a final 10 min extension at 72°C. The resulting product was sequenced with Sanger sequencing, and colonies identified as *Apilactobacillus kunkeei* were once again isolated and placed in 10 µL of MilliQ (EMD Millipore) water. The variable segments of three housekeeping genes, *lepA*, *recG*, and *rpoB* were amplified using previously described primers [14, 15]. The 25 µL PCR amplification was made up of 11 µL of PCR-grade water, 12 µL of MyTaq Red Mix (Bioline), 0.5 µL of each 10 µM forward and reverse primer, and 1 µL of the diluted colony. PCR conditions followed those for the 16S rRNA gene above except that the annealing temperature was 47°C for *lepA* and *recG*, and 60°C for *rpoB.* [14, 15].

The amplified PCR product was sequenced with Sanger sequencing. As previously described [16], the sequences were aligned with MAFFT [17] and trimmed with TrimAI [18] with a penalty for more than 50% gaps. Individual gene trees and the partitioned analysis for the multi-gene alignment were inferred using IQ-TREE [16, 19]. Statistical support for the phylogenies was calculated using 1000 replicates of both a Shimodaira-Hasegawa-like approximation likelihood test and ultrafast bootstrapping along with a Bayesian-like transformation of aLRT [16, 20, 21]. The partition model was selected by the IQ-TREE ModelFinder (Table S3) [16, 22]. *Apilactobacillus apinorum* Fhon13 (Table S4) [16] served as the outgroup for this phylogenetic tree. Outlier long branches were trimmed with TreeShrink [23].

**Fig S1**. Pairwise sample differential abundance based on DESeq2 analysis. The four panels show comparisons of **(a)** winter crop and winter mouth, **(b)** winter nectar and winter mouth, **(c)** winter nectar and winter crop, and **(d)** summer crop and summer mouth. Only statistically significant differences are plotted and colored by Phylum. Taxa are agglomerated to and labeled by Genus

sample type

*Apis cerana*

*Apis mellifera*

b

a

**Fig S2.** Samples collected from *Apis cerana* and *Apis mellifera* sites show the same overall pattern of more common and constant ASVs_97_ in the crop than the mouth and the nectar. (**a**) At *Apis cerana* sites, only 8% and 4% of the winter crop ASVs_97_ were shared with the mouth and nectar samples, respectively, while the mouth and nectar shared about 30% of their ASVs_97_. (**b**) Similarly, at *Apis mellifera* sites, 6 and 9% of the crop ASVs_97_ were shared with the mouth and nectar samples, respectively. Half of the mouth ASVs_97_ were also present in the nectar, while only about 30% of the nectar ASVs_97_ were found in the mouth. Venn diagrams are colored by sample type with ASVs_97_ counts in bold as in Figure 2

**Fig S3.** Heatmap as in Fig. 6 except that samples are ungrouped and the heatmap represents presence (in green) and absence (in black) of the taxa in each sample. Also, all differentially abundant taxa (see Supplementary Figure 1), agglomerated to genus, are depicted.


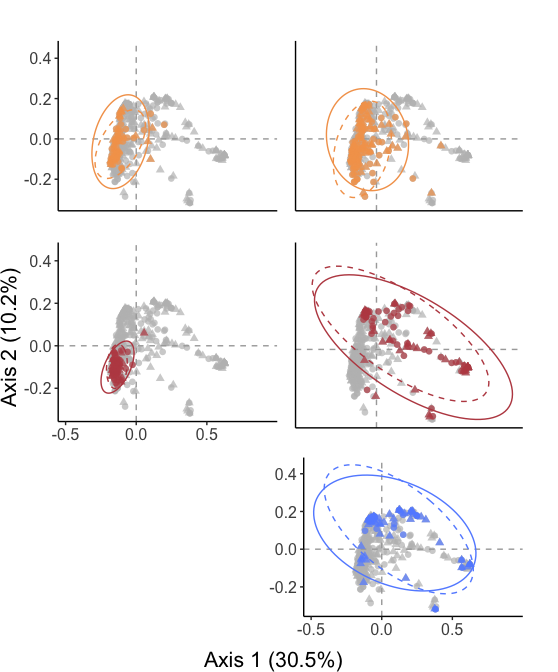

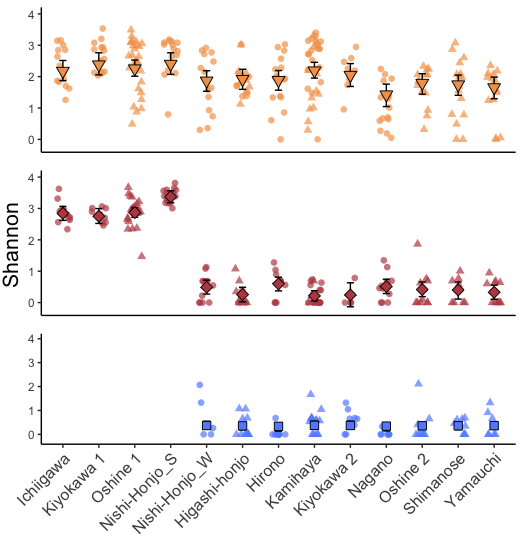


a

b

**Fig S4.** Exclusion of genera commonly found in the hindgut, specifically *Gilliamella, Snodgrassella,* and *Lactobacillus*, does not change the (**a**) alpha or (**b**) beta diversity trends of the bacterial communities. The crop bacterial alpha diversity and composition remain more constant across season than those of the mouth, while the mouth and nectar present very similar alpha and beta diversity in the winter.

Summer

Winter

Summer

Winter


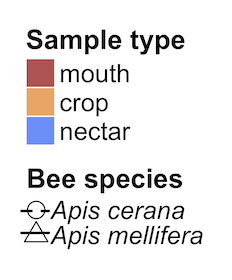


winter

summer

**Fig S5.** Median coefficients with error bars representing the bootstrapped 95% confidence interval of linear mixed effects model of Shannon diversity with the fixed effects of season and bee species, and the random effect of site.


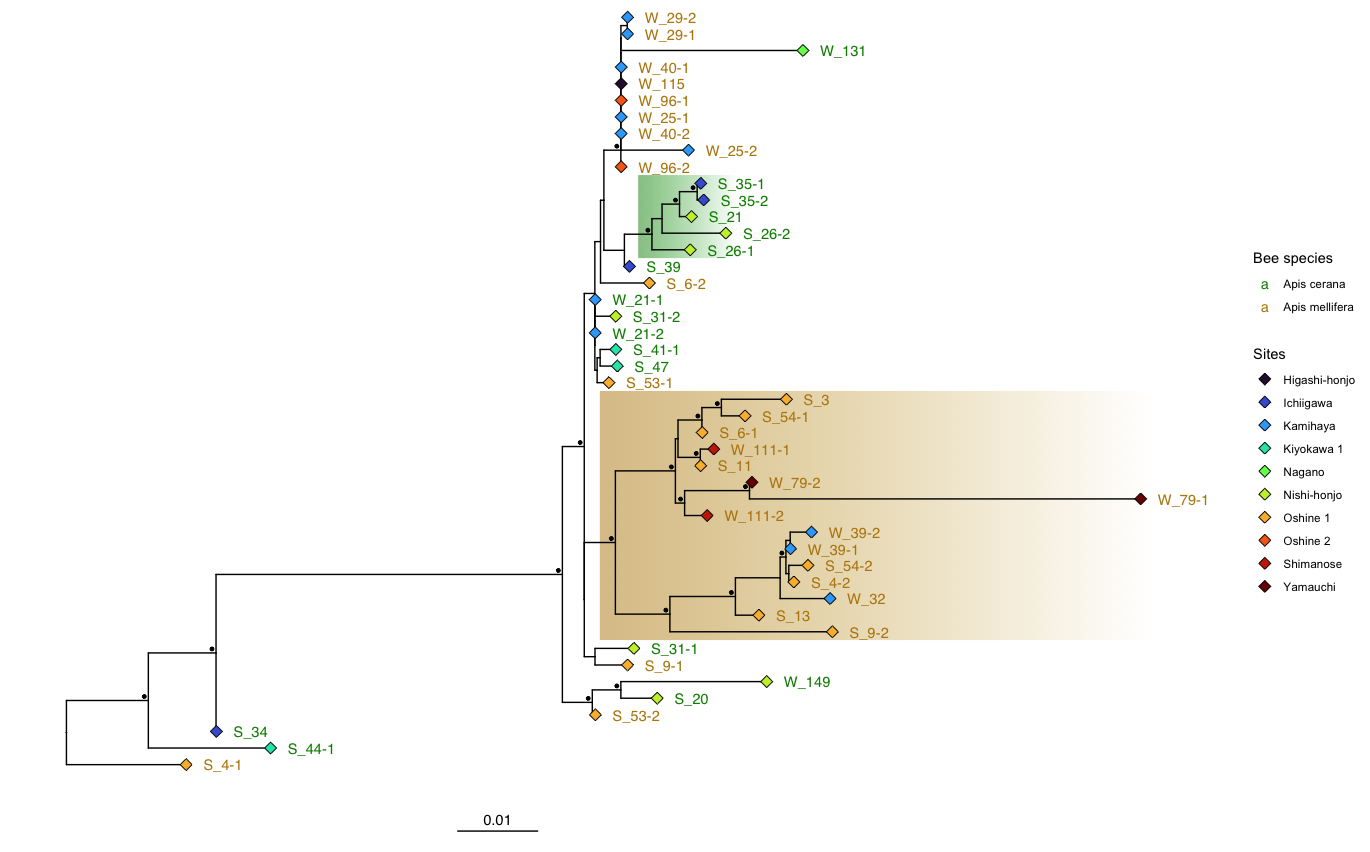


*Apis mellifera*

*Apis cerana*

b

a

**Fig S6.** (**a**) Cladogram of phylogeny as in Fig. 8 including the outgroup, *Apilactobacillus apinorum* Fhon13, and statistical support from 1000 SH-aLRT pseudo replicates (%), a Bayesian-like transformation of aLRT (aBayes), and 1000 ultrafast bootstraps (%) in that order. (**b**) Multi-gene phylogeny including Fhon13. Branch length represents substitution frequency. Nodes are colored by collection site; sample labels start with an “S” for summer or a “W” for winter samples followed by the host bee ID and the isolate number and are colored by host species.

**Figure S7.** Melting curves from the qPCR assays.

**Figure S8.** ASV accumulation curves for (**a**) crop, (**b**) mouth, and (**c**) nectar samples. The colors represent the different samples, and the lines are coded to show the sample-size-based rarefaction or extrapolation sampling curves.

**Data S1**. Data table of apricot orchard collection year, season, latitude, and longitude.

| **year** | **season** | **location** | **latitude** | **longitude** |
| --- | --- | --- | --- | --- |
| 2018 | summer | Oshine 1 | 33.789 | 135.34 |
| 2018 | both | Nishi-Honjo | 33.796 | 135.328 |
| 2018 | summer | Ichiigawa | 33.858 | 135.349 |
| 2018 | summer | Kiyokawa 1 | 33.844 | 135.385 |
| 2019 | winter | Kamihaya | 33.823 | 135.408 |
| 2019 | winter | Kiyokawa 2 | 33.861 | 135.403 |
| 2019 | both | Nishi-Honjo | 33.796 | 135.328 |
| 2019 | winter | Hirono | 33.848 | 135.358 |
| 2019 | winter | Nagano | 33.766 | 135.435 |
| 2019 | winter | Oshine 2 | 33.794 | 135.34 |
| 2019 | winter | Yamauchi | 33.77 | 135.301 |
| 2019 | winter | Shimanose | 33.831 | 135.362 |
| 2019 | winter | Higashi-Honjo | 33.819 | 135.353 |

**Data S2**. Data table with quantitative PCR values and sample type for each sample where the 16S rRNA gene was quantified.

| **samples** | **type** | **quantification** | **log quantification** |
| --- | --- | --- | --- |
| JB_S_B2_C15 | crop | 1352670.59 | 6.13119205 |
| JB_S_B1_M15 | mouth | 22003.4836 | 4.34249145 |
| JB_S_B1_C26 | crop | 62027.6068 | 4.79258503 |
| JB_S_B2_C16 | crop | 50253.5125 | 4.70116642 |
| JB_S_B1_M01 | mouth | 28576.8245 | 4.45601397 |
| JB_S_B1_M13 | mouth | 20483.9223 | 4.31141312 |
| JB_S_B1_C03 | crop | 103353.511 | 5.01432523 |
| JB_S_B1_C17 | crop | 56762.7945 | 4.75406377 |
| JB_S_B1_C56 | crop | 75937.4119 | 4.88045579 |
| JB_S_B2_C28 | crop | 42179.2346 | 4.62509869 |
| JB_S_B1_C04 | crop | 61912.0323 | 4.79177506 |
| JB_S_B2_C29 | crop | 37615.7914 | 4.5753702 |
| JB_S_B1_C05 | crop | 85804.4795 | 4.93350996 |
| JB_S_B1_C19 | crop | 34306.7453 | 4.53537952 |
| JB_S_B1_M17 | mouth | 28662.3991 | 4.45731254 |
| JB_S_B2_C30 | crop | 20615.2941 | 4.31418954 |
| JB_S_B2_C25 | crop | 56724.5218 | 4.75377084 |
| JB_S_B1_C20 | crop | 111387.923 | 5.04683811 |
| JB_S_B1_C24 | crop | 108052.548 | 5.03363501 |
| JB_S_B2_C13 | crop | 61402.4849 | 4.78818595 |
| JB_S_B1_C53 | crop | 158226.051 | 5.19927799 |
| JB_S_B1_C23 | crop | 77170.2179 | 4.88744973 |
| JB_S_B1_C52 | crop | 147390.375 | 5.16846912 |
| JB_S_B1_C22 | crop | 43626.7865 | 4.63975323 |
| JB_S_B2_C11 | crop | 31922.1197 | 4.50409172 |
| JB_S_B1_C51 | crop | 11096.6474 | 4.04519179 |
| JB_S_B1_M05 | mouth | 6361.72281 | 3.80357474 |
| JB_S_B1_M11 | mouth | 7904.97305 | 3.89790039 |
| JB_S_B1_C21 | crop | 38731.8601 | 4.58806836 |
| JB_S_B2_C32 | crop | 25085.9476 | 4.39943051 |
| JB_S_B1_C50 | crop | 55492.1546 | 4.74423159 |
| JB_S_B1_M04 | mouth | 22948.4917 | 4.36075415 |
| JB_S_B2_M51 | mouth | 10143.5851 | 4.00619148 |
| JB_S_B2_C09 | crop | 41915.4486 | 4.62237412 |
| JB_S_B2_C31 | crop | 45349.5616 | 4.65657309 |
| JB_S_B1_M03 | mouth | 10499.4019 | 4.02116456 |
| JB_S_B1_C01 | crop | 25808.7071 | 4.41176625 |
| JB_S_B2_M26 | mouth | 11955.5432 | 4.07756931 |
| 2019_JB_W_B2_M010 | mouth | 11454.0712 | 4.05895988 |
| JB_S_B2_M50 | mouth | 12170.4825 | 4.0853078 |
| JB_S_B1_M16 | mouth | 8944.73519 | 3.95156749 |
| JB_S_B2_M28 | mouth | 8167.15349 | 3.91207072 |
| JB_S_B1_M09 | mouth | 13293.6793 | 4.1236452 |
| JB_S_B2_M52 | mouth | 12397.5539 | 4.09333601 |
| JB_S_B2_M29 | mouth | 10580.6768 | 4.02451345 |
| JB_S_B2_M25 | mouth | 4055.08345 | 3.6079998 |
| JB_S_B2_M53 | mouth | 15867.6945 | 4.20051383 |
| JB_S_B2_M30 | mouth | 4907.95266 | 3.69090037 |
| JB_S_B2_M24 | mouth | 9908.49252 | 3.99600759 |
| JB_S_B2_M31 | mouth | 11264.0461 | 4.05169442 |
| JB_S_B2_M23 | mouth | 11257.7261 | 4.05145068 |
| JB_S_B2_M22 | mouth | 7669.86157 | 3.88478753 |
| JB_S_B2_M21 | mouth | 12364.3887 | 4.09217265 |
| JB_S_B2_M56 | mouth | 18558.6157 | 4.26854558 |
| JB_S_B1_M20 | mouth | 16233.7957 | 4.21042008 |
| JB_S_B2_M19 | mouth | 17670.0905 | 4.24723878 |
| JB_S_B2_M32 | mouth | 9448.91757 | 3.97538206 |
| JB_W_B1_10 | crop | 81879.9404 | 4.91317752 |
| JB_W_B1_23 | crop | 18256.3196 | 4.26141323 |
| JB_W_B1_28 | crop | 5123.12559 | 3.709535 |
| JB_W_B1_38 | crop | 5205.56939 | 3.71646824 |
| JB_W_B1_74 | crop | 1570.62208 | 3.1960717 |
| JB_W_B1_83 | crop | 2137.26893 | 3.32985917 |
| JB_W_B1_81 | crop | 7336.96223 | 3.86551628 |
| JB_W_B1_80 | crop | 8406.64801 | 3.92462286 |
| JB_W_B1_79 | crop | 9893.51593 | 3.99535066 |
| JB_W_B2_92 | crop | 8328.14024 | 3.92054803 |
| JB_W_B2_100 | crop | 7443.18634 | 3.87175889 |
| JB_W_B2_99 | crop | 1573.00362 | 3.19672972 |
| JB_W_B2_94 | crop | 9918.07924 | 3.99642757 |
| JB_W_B2_105 | crop | 36839.0127 | 4.56630798 |
| JB_W_B2_115 | crop | 67120.9661 | 4.8268582 |
| JB_W_B2_114 | crop | 6111.67103 | 3.78615997 |
| JB_W_B2_147 | crop | 3239.35959 | 3.51045916 |
| JB_W_B2_146 | crop | 3366.76923 | 3.52721335 |
| JB_W_B2_155 | crop | 30831.3416 | 4.48899242 |
| JB_W_B2_154 | crop | 33974.5276 | 4.53115343 |
| JB_W_B2_153 | crop | 2320.17085 | 3.36551997 |
| JB_W_B2_152 | crop | 98882.3503 | 4.99511878 |
| JB_W_B2_151 | crop | 20519.4022 | 4.3121647 |
| JB_W_B2_150 | crop | 57158.9682 | 4.75708438 |
| JB_W_B2_149 | crop | 210237.16 | 5.32270948 |
| JB_W_B2_159 | crop | 18499.852 | 4.26716826 |
| JB_W_B2_157 | crop | 11902.4695 | 4.07563708 |
| JB_W_B2_156 | crop | 60108.6846 | 4.77893722 |
| 2019_JB_W_B1_M094 | mouth | 12636.4089 | 4.10162367 |
| 2019_JB_W_B1_M092 | mouth | 17770.8221 | 4.24970752 |
| 2019_JB_W_B1_M152 | mouth | 9795.97458 | 3.99104765 |
| 2019_JB_W_B1_M151 | mouth | 8227.12784 | 3.91524825 |
| 2019_JB_W_B1_M150 | mouth | 10136.8969 | 4.00590503 |
| 2019_JB_W_B1_M149 | mouth | 9265.98529 | 3.96689161 |
| 2019_JB_W_B1_M147 | mouth | 10415.6203 | 4.01768514 |
| 2019_JB_W_B1_M146 | mouth | 12629.7348 | 4.10139423 |
| 2019_JB_W_B1_M080 | mouth | 11617.3508 | 4.06510711 |
| 2019_JB_W_B1_M079 | mouth | 10983.8938 | 4.04075633 |
| 2019_JB_W_B1_M074 | mouth | 9663.42304 | 3.98513099 |
| 2019_JB_W_B1_M083 | mouth | 11056.8528 | 4.04363153 |
| 2019_JB_W_B1_M081 | mouth | 12826.936 | 4.10812293 |
| 2019_JB_W_B1_M038 | mouth | 10609.2469 | 4.02568456 |
| 2019_JB_W_B1_M115 | mouth | 4391.96328 | 3.6426587 |
| 2019_JB_W_B1_M114 | mouth | 7104.81488 | 3.85155277 |
| 2019_JB_W_B2_M023 | mouth | 13285.3105 | 4.12337171 |
| 2019_JB_W_B2_M028 | mouth | 22124.3849 | 4.34487121 |
| 2019_JB_W_B2_M100 | mouth | 14327.7888 | 4.15617917 |
| 2019_JB_W_B2_M099 | mouth | 20457.433 | 4.31085114 |
| 2019_JB_W_B2_M105 | mouth | 12476.8189 | 4.09610387 |
| 2019_JB_W_B2_M159 | mouth | 9412.42917 | 3.97370172 |
| 2019_JB_W_B2_M157 | mouth | 23787.8563 | 4.37635531 |
| 2019_JB_W_B2_M156 | mouth | 12933.5717 | 4.11171848 |
| 2019_JB_W_B2_M155 | mouth | 23085.3502 | 4.36333647 |
| 2019_JB_W_B2_M154 | mouth | 19715.7209 | 4.29481266 |
| 2019_JB_W_B2_M153 | mouth | 12009.1163 | 4.07951105 |

**Data S3**. Data table with the name of each *Apilactobacillus kunkeei* isolate used in the phylogeny (see Fig. 8 and S6), along with the species and site it was isolated from, and the tag used in the phylogenetic tree.

| **sample** | **bee species** | **site** | **isolate** | **tag** |
| --- | --- | --- | --- | --- |
| Fhon13 | mellifera | Sweden | Fhon13 | Fhon13 |
| W_21-1 | cerana | Kamihaya | W_21.1 | WC21.1 |
| W_21-2 | cerana | Kamihaya | W_21.2 | WC21.2 |
| W_25-1 | mellifera | Kamihaya | W_25.1 | WM25.1 |
| W_25-2 | mellifera | Kamihaya | W_25.2 | WM25.2 |
| W_29-1 | mellifera | Kamihaya | W_29.1 | WM29.1 |
| W_29-2 | mellifera | Kamihaya | W_29.2 | WM29.2 |
| W_32-1 | mellifera | Kamihaya | W_32 | WM32 |
| W_39-1 | mellifera | Kamihaya | W_39.1 | WM39.1 |
| W_39-2 | mellifera | Kamihaya | W_39.2 | WM39.2 |
| W_40-1 | mellifera | Kamihaya | W_40.1 | WM40.1 |
| W_40-2 | mellifera | Kamihaya | W_40.2 | WM40.2 |
| W_79-1 | mellifera | Yamauchi | W_79.1 | WM79.1 |
| W_79-2 | mellifera | Yamauchi | W_79.2 | WM79.2 |
| W_96-1 | mellifera | Oshine 2 | W_96.1 | WM96.1 |
| W_96-2 | mellifera | Oshine 2 | W_96.2 | WM96.2 |
| W_111-1 | mellifera | Shimanose | W_111.1 | WM111.1 |
| W_111-2 | mellifera | Shimanose | W_111.2 | WM111.2 |
| W_115-1 | mellifera | Higashi-Honjo | W_115 | WM115 |
| W_131-1 | cerana | Nagano | W_131 | WC131 |
| W_149-1 | cerana | Nishi-Honjo | W_149 | WC149 |
| S_1 | mellifera | Oshine 1 | S_13 | SM13 |
| S_2 | cerana | Nishi-Honjo | S_31.1 | SC31.1 |
| S_3 | cerana | Nishi-Honjo | S_20 | SC20 |
| S_4 | cerana | Nishi-Honjo | S_31.2 | SC31.2 |
| S_5 | mellifera | Oshine 1 | S_4.1 | SM4.1 |
| S_6 | mellifera | Oshine 1 | S_4.2 | SM4.2 |
| S_8 | mellifera | Oshine 1 | S_3 | SM3 |
| S_9 | cerana | Nishi-Honjo | S_21 | SC21 |
| S_10 | cerana | Nishi-Honjo | S_26.1 | SC26.1 |
| S_11 | mellifera | Oshine 1 | S_5 | SM5 |
| S_13 | cerana | Nishi-Honjo | S_23 | SC23 |
| S_14 | mellifera | Oshine 1 | S_6.1 | SM6.1 |
| S_19 | mellifera | Oshine 1 | S_9.1 | SM9.1 |
| S_20 | mellifera | Oshine 1 | S_9.2 | SM9.2 |
| S_23 | mellifera | Oshine 1 | S_6.2 | SM6.2 |
| S_27 | cerana | Kiyokawa 1 | S_44.1 | SC44.1 |
| S_29 | cerana | Ichiigawa | S_34 | SC34 |
| S_31 | cerana | Ichiigawa | S_35.1 | SC35.1 |
| S_32 | cerana | Ichiigawa | S_35.2 | SC35.2 |
| S_33 | mellifera | Oshine 1 | S_54.1 | SM54.1 |
| S_34 | mellifera | Oshine 1 | S_53.1 | SM53.1 |
| S_46 | cerana | Kiyokawa 1 | S_47 | SC47 |
| S_47 | cerana | Kiyokawa 1 | S_41.1 | SC41.1 |
| S_60 | cerana | Ichiigawa | S_39 | SC39 |
| S_62 | mellifera | Oshine 1 | S_53.2 | SM53.2 |
| S_64 | mellifera | Oshine 1 | S_54.2 | SM54.2 |
| S_86 | mellifera | Oshine 1 | S_11 | SM11 |
| S_115 | cerana | Nishi-Honjo | S_26.2 | SC26.2 |

**Table S1**. Summary of the bees used in this study.

| **date** | **season** | **site** | **latitude** | **longitude** | **bee species** | **bee count** |
| --- | --- | --- | --- | --- | --- | --- |
| 2018-08-02 | summer | Nishi-Honjo | 33.796 | 135.328 | *A. cerana* | 16 |
| 2019-02-25 | winter | Nishi-Honjo | 33.796 | 135.328 | *A. cerana* | 14 |
| 2018-08-03 | summer | Ichiigawa | 33.858 | 135.349 | *A. cerana* | 14 |
| 2018-08-03 | summer | Kiyokawa 1 | 33.844 | 135.385 | *A. cerana* | 14 |
| 2018-08-02 | summer | Oshine 1 | 33.789 | 135.34 | *A. mellifera* | 28 |
| 2019-02-21 | winter | Kamihaya | 33.823 | 135.408 | *A. cerana* | 20 |
| 2019-02-22 | winter | Hirono | 33.848 | 135.358 | *A. cerana* | 16 |
| 2019-02-25 | winter | Nagano | 33.766 | 135.435 | *A. cerana* | 16 |
| 2019-02-22 | winter | Kiyokawa 2 | 33.861 | 135.403 | *A. cerana* | 8 |
| 2019-02-24 | winter | Higashi-Honjo | 33.819 | 135.353 | *A. cerana* | 8 |
| 2019-02-23 | winter | Yamauchi | 33.77 | 135.301 | *A. mellifera* | 16 |
| 2019-02-24 | winter | Shimanose | 33.831 | 135.362 | *A. mellifera* | 16 |
| 2019-02-23 | winter | Oshine 2 | 33.794 | 135.34 | *A. mellifera* | 15 |
| 2019-02-21 | winter | Kamihaya | 33.823 | 135.408 | *A. mellifera* | 12 |
| 2019-02-24 | winter | Higashi-Honjo | 33.819 | 135.353 | *A. mellifera* | 8 |

**Table S2**. Sample type group IDs and their corresponding attributes.

| **group** | **type** | **season** | **Apis sp.** | **site** |
| --- | --- | --- | --- | --- |
| C1 | crop | summer | cerana | Ichiigawa |
| C2 | crop | summer | cerana | Kiyokawa 1 |
| C3 | crop | summer | cerana | Nishi-Honjo |
| C4 | crop | summer | mellifera | Oshine 1 |
| C5 | crop | winter | cerana | Nishi-Honjo |
| C6 | crop | winter | cerana | Higashi-Honjo |
| C7 | crop | winter | cerana | Hirono |
| C8 | crop | winter | cerana | Kamihaya |
| C9 | crop | winter | cerana | Kiyokawa 2 |
| C10 | crop | winter | cerana | Nagano |
| C11 | crop | winter | mellifera | Higashi-Honjo |
| C12 | crop | winter | mellifera | Kamihaya |
| C13 | crop | winter | mellifera | Oshine 2 |
| C14 | crop | winter | mellifera | Shimanose |
| C15 | crop | winter | mellifera | Yamauchi |
| M1 | mouth | summer | cerana | Ichiigawa |
| M2 | mouth | summer | cerana | Kiyokawa 1 |
| M3 | mouth | summer | cerana | Nishi-Honjo |
| M4 | mouth | summer | mellifera | Oshine 1 |
| M5 | mouth | winter | cerana | Nishi-Honjo |
| M6 | mouth | winter | cerana | Higashi-Honjo |
| M7 | mouth | winter | cerana | Hirono |
| M8 | mouth | winter | cerana | Kamihaya |
| M9 | mouth | winter | cerana | Kiyokawa 2 |
| M10 | mouth | winter | cerana | Nagano |
| M11 | mouth | winter | mellifera | Higashi-Honjo |
| M12 | mouth | winter | mellifera | Kamihaya |
| M13 | mouth | winter | mellifera | Oshine 2 |
| M14 | mouth | winter | mellifera | Shimanose |
| M15 | mouth | winter | mellifera | Yamauchi |
| N1 | nectar | winter | cerana | Nishi-Honjo |
| N2 | nectar | winter | cerana | Hirono |
| N3 | nectar | winter | cerana | Kiyokawa 2 |
| N4 | nectar | winter | cerana | Nagano |
| N5 | nectar | winter | mellifera | Higashi-Honjo |
| N6 | nectar | winter | mellifera | Kamihaya |
| N7 | nectar | winter | mellifera | Oshine 2 |
| N8 | nectar | winter | mellifera | Shimanose |
| N9 | nectar | winter | mellifera | Yamauchi |

**Table S3**. Partition models chosen by IQ-TREE ModelFinder

| **partition** | **model** |
| --- | --- |
| 16S rRNA | TIM2e + I + R2 |
| lepA1 | TIM2 + F + R2 |
| lepA2 | TPM2u + F + R2 |
| recG | TN + F + R2 |
| rpoB | TN + F + R2 |

**Table S4**. Genbank accession numbers for all strains used in *Apilactobacillus kunkeei* phylogeny (see Figures 7 and S7). Where a high-quality sequence for a gene could not be obtained the gene’s accession number is labeled with "NA".

| **species** | **isolate** | **16S rRNA** | ***lepA1*** | ***lepA2*** | ***recG*** | ***rpoB*** | **Reference** |
| --- | --- | --- | --- | --- | --- | --- | --- |
| *A. apinorum* | Fhon13 | OX335302.1 | OX335302.1 | OX335302.1 | OX335302.1 | OX335302.1 | Tamarit et al. 2015 |
| *A. kunkeei* | W_21.1 | PP341993 | PP382315 | PP382266 | PP382363 | PP412473 | This study |
| *A. kunkeei* | W_21.2 | PP341994 | PP382316 | PP382267 | PP382364 | PP412474 | This study |
| *A. kunkeei* | W_25.1 | PP341995 | PP382317 | PP382268 | PP382365 | PP412475 | This study |
| *A. kunkeei* | W_25.2 | PP341996 | PP382318 | PP382269 | PP382366 | PP412476 | This study |
| *A. kunkeei* | W_29.1 | PP341997 | PP382319 | PP382270 | PP382367 | PP412477 | This study |
| *A. kunkeei* | W_29.2 | PP341998 | PP382320 | PP382271 | PP382368 | PP412478 | This study |
| *A. kunkeei* | W_32.1 | PP341999 | PP382321 | PP382272 | PP382369 | PP412479 | This study |
| *A. kunkeei* | W_39.1 | PP342000 | PP382322 | PP382273 | PP382370 | PP412480 | This study |
| *A. kunkeei* | W_39.2 | PP342001 | PP382323 | PP382274 | PP382371 | PP412481 | This study |
| *A. kunkeei* | W_40.1 | PP342002 | PP382324 | PP382275 | PP382372 | PP412482 | This study |
| *A. kunkeei* | W_40.2 | PP342003 | PP382325 | PP382276 | PP382373 | PP412483 | This study |
| *A. kunkeei* | W_79.1 | PP342004 | PP382326 | PP382277 | PP382374 | PP412484 | This study |
| *A. kunkeei* | W_79.2 | PP342005 | PP382327 | PP382278 | PP382375 | NA | This study |
| *A. kunkeei* | W_96.1 | PP342006 | PP382328 | PP382279 | PP382376 | PP412485 | This study |
| *A. kunkeei* | W_96.2 | PP342007 | NA | PP382280 | PP382377 | PP412486 | This study |
| *A. kunkeei* | W_111.1 | PP342008 | PP382329 | PP382281 | PP382378 | PP412487 | This study |
| *A. kunkeei* | W_111.2 | PP342009 | PP382330 | PP382282 | PP382379 | PP412488 | This study |
| *A. kunkeei* | W_115.1 | PP342010 | PP382331 | PP382283 | PP382380 | PP412489 | This study |
| *A. kunkeei* | W_131.1 | NA | PP382332 | PP382284 | NA | PP412490 | This study |
| *A. kunkeei* | W_149.1 | PP342011 | PP382333 | PP382285 | PP382381 | PP412491 | This study |
| *A. kunkeei* | S_1 | NA | PP382334 | PP382286 | PP382382 | NA | This study |
| *A. kunkeei* | S_2 | PP342012 | PP382335 | PP382287 | PP382383 | PP412492 | This study |
| *A. kunkeei* | S_3 | PP342013 | PP382336 | PP382288 | PP382384 | PP412493 | This study |
| *A. kunkeei* | S_4 | PP342014 | PP382337 | PP382289 | PP382385 | PP412494 | This study |
| *A. kunkeei* | S_5 | PP342015 | PP382338 | PP382290 | NA | PP412495 | This study |
| *A. kunkeei* | S_6 | PP342016 | PP382339 | PP382291 | PP382386 | PP412496 | This study |
| *A. kunkeei* | S_8 | PP342017 | PP382340 | PP382292 | PP382387 | PP412497 | This study |
| *A. kunkeei* | S_9 | PP342018 | PP382341 | PP382293 | PP382388 | PP412498 | This study |
| *A. kunkeei* | S_10 | PP342019 | PP382342 | PP382294 | PP382389 | PP412499 | This study |
| *A. kunkeei* | S_11 | PP342020 | PP382343 | PP382295 | PP382390 | PP412500 | This study |
| *A. kunkeei* | S_13 | PP342021 | PP382344 | PP382296 | PP382391 | PP412501 | This study |
| *A. kunkeei* | S_14 | PP342022 | PP382345 | PP382297 | PP382392 | PP412502 | This study |
| *A. kunkeei* | S_19 | PP342023 | PP382346 | PP382298 | PP382393 | PP412503 | This study |
| *A. kunkeei* | S_20 | PP342024 | PP382347 | PP382299 | PP382394 | PP412504 | This study |
| *A. kunkeei* | S_23 | PP342025 | PP382348 | PP382300 | PP382395 | PP412505 | This study |
| *A. kunkeei* | S_27 | PP342026 | PP382349 | PP382301 | PP382396 | PP412506 | This study |
| *A. kunkeei* | S_29 | PP342027 | PP382350 | PP382302 | PP382397 | PP412507 | This study |
| *A. kunkeei* | S_31 | NA | PP382351 | PP382303 | PP382398 | PP412508 | This study |
| *A. kunkeei* | S_32 | PP342028 | PP382352 | PP382304 | PP382399 | PP412509 | This study |
| *A. kunkeei* | S_33 | PP342029 | PP382353 | PP382305 | PP382400 | PP412510 | This study |
| *A. kunkeei* | S_34 | PP342030 | PP382354 | PP382306 | PP382401 | PP412511 | This study |
| *A. kunkeei* | S_46 | PP342031 | PP382355 | PP382307 | PP382402 | PP412512 | This study |
| *A. kunkeei* | S_47 | PP342032 | PP382356 | PP382308 | PP382403 | PP412513 | This study |
| *A. kunkeei* | S_48 | PP342033 | PP382357 | PP382309 | PP382404 | PP412514 | This study |
| *A. kunkeei* | S_60 | PP342034 | PP382358 | PP382310 | PP382405 | PP412515 | This study |
| *A. kunkeei* | S_62 | PP342035 | PP382359 | PP382311 | PP382406 | PP412516 | This study |
| *A. kunkeei* | S_64 | PP342036 | PP382360 | PP382312 | PP382407 | PP412517 | This study |
| *A. kunkeei* | S_86 | PP342037 | PP382361 | PP382313 | PP382408 | PP412518 | This study |
| *A. kunkeei* | S_115 | PP342038 | PP382362 | PP382314 | PP382409 | PP412519 | This study |

**Table S5.** Quantitative PCR (qPCR) standard curve information.

|  | Efficiency  (%) | R^2^  value |  | Efficiency  (%) | R^2^  value |  | Efficiency  (%) | R^2^  value |
| --- | --- | --- | --- | --- | --- | --- | --- | --- |
| Run #1 | 93.796 | 0.996 | Run #21 | 90.432 | 0.996 | Run #41 | 93.364 | 0.986 |
| Run #2 | 90.956 | 0.995 | Run #22 | 91.553 | 0.994 | Run #42 | 92.516 | 0.999 |
| Run #3 | 92.309 | 0.996 | Run #23 | 90.836 | 0.998 | Run #43 | 90.414 | 0.999 |
| Run #4 | 90.729 | 0.997 | Run #24 | 90.189 | 0.994 | Run #44 | 93.091 | 0.998 |
| Run #5 | 92.241 | 0.998 | Run #25 | 96.353 | 0.995 | Run #45 | 90.315 | 0.997 |
| Run #6 | 98.303 | 0.995 | Run #26 | 94.487 | 0.996 | Run #46 | 92.153 | 0.999 |
| Run #7 | 98.976 | 0.996 | Run #27 | 90.439 | 0.993 | Run #47 | 90.992 | 0.999 |
| Run #8 | 99.181 | 0.996 | Run #28 | 96.085 | 0.997 | Run #48 | 96.834 | 0.992 |
| Run #9 | 92.710 | 0.989 | Run #29 | 96.285 | 0.995 | Run #49 | 91.589 | 0.997 |
| Run #10 | 90.953 | 0.986 | Run #30 | 97.259 | 0.998 | Run #50 | 95.543 | 0.985 |
| Run #11 | 90.221 | 0.991 | Run #31 | 96.913 | 0.990 | Run #51 | 95.434 | 0.990 |
| Run #12 | 95.103 | 0.982 | Run #32 | 96.221 | 0.994 | Run #52 | 97.988 | 0.988 |
| Run #13 | 94.940 | 0.995 | Run #33 | 90.003 | 0.999 | Run #53 | 95.646 | 0.988 |
| Run #14 | 92.124 | 0.993 | Run #34 | 92.481 | 0.995 | Run #54 | 91.150 | 0.997 |
| Run #15 | 87.615 | 0.999 | Run #35 | 90.894 | 0.996 | Run #55 | 94.581 | 0.996 |
| Run #16 | 94.205 | 0.998 | Run #36 | 93.426 | 0.995 |  |  |  |
| Run #17 | 91.311 | 0.998 | Run #37 | 93.346 | 0.996 |  |  |  |
| Run #18 | 94.559 | 0.998 | Run #38 | 97.959 | 0.993 |  |  |  |
| Run #19 | 90.964 | 0.992 | Run #39 | 93.292 | 0.991 |  |  |  |
| Run #20 | 95.475 | 0.990 | Run #40 | 97.411 | 0.994 |  |  |  |

**Table S6.** Summary of DADA2 processing read counts for each sample.

| **sample** | **input** | **filtered** | **dada** | **merged** | **non-**  **chimeric** | **percent**  **reads**  **retained** |
| --- | --- | --- | --- | --- | --- | --- |
| JB-S-B1-C01 | 38569 | 33873 | 33726 | 33356 | 33280 | 86.3 |
| JB-S-B1-C02 | 44854 | 38747 | 38680 | 38388 | 38330 | 85.5 |
| JB-S-B1-C03 | 47262 | 41466 | 41304 | 41043 | 40882 | 86.5 |
| JB-S-B1-C04 | 55272 | 48649 | 48497 | 47951 | 47528 | 86 |
| JB-S-B1-C05 | 38067 | 32896 | 32806 | 32347 | 32229 | 84.7 |
| JB-S-B1-C06 | 39768 | 35560 | 35450 | 34909 | 34742 | 87.4 |
| JB-S-B1-C07 | 45720 | 40272 | 40194 | 40076 | 40066 | 87.6 |
| JB-S-B1-C08 | 42322 | 36465 | 36381 | 36049 | 36004 | 85.1 |
| JB-S-B1-C17 | 31584 | 27614 | 27530 | 27371 | 27332 | 86.5 |
| JB-S-B1-C18 | 52636 | 46143 | 46055 | 45573 | 45537 | 86.5 |
| JB-S-B1-C19 | 43709 | 38549 | 38456 | 38132 | 38051 | 87.1 |
| JB-S-B1-C20 | 51824 | 46147 | 46000 | 45567 | 45283 | 87.4 |
| JB-S-B1-C21 | 32111 | 20307 | 20208 | 19999 | 19819 | 61.7 |
| JB-S-B1-C22 | 31243 | 26860 | 26773 | 26427 | 26328 | 84.3 |
| JB-S-B1-C23 | 48486 | 41128 | 40996 | 40456 | 40340 | 83.2 |
| JB-S-B1-C24 | 30343 | 26218 | 26093 | 25777 | 25613 | 84.4 |
| JB-S-B1-C26 | 42417 | 38055 | 37939 | 37046 | 36970 | 87.2 |
| JB-S-B1-C33 | 34151 | 28497 | 28353 | 28195 | 28100 | 82.3 |
| JB-S-B1-C34 | 51130 | 44949 | 44801 | 44156 | 43624 | 85.3 |
| JB-S-B1-C35 | 31326 | 27249 | 27178 | 26985 | 26958 | 86.1 |
| JB-S-B1-C36 | 55024 | 48907 | 48757 | 48184 | 47834 | 86.9 |
| JB-S-B1-C37 | 52951 | 45823 | 45702 | 45225 | 45026 | 85 |
| JB-S-B1-C38 | 20405 | 13948 | 13879 | 13636 | 13521 | 66.3 |
| JB-S-B1-C39 | 36593 | 31916 | 31860 | 31754 | 31734 | 86.7 |
| JB-S-B1-C40 | 33260 | 28852 | 28797 | 28642 | 28522 | 85.8 |
| JB-S-B1-C48 | 39739 | 35710 | 35484 | 34979 | 34951 | 88 |
| JB-S-B1-C49 | 31561 | 28450 | 28370 | 28053 | 27611 | 87.5 |
| JB-S-B1-C50 | 27351 | 23799 | 23750 | 23435 | 23403 | 85.6 |
| JB-S-B1-C51 | 18703 | 15918 | 15861 | 15583 | 15583 | 83.3 |
| JB-S-B1-C52 | 32194 | 28343 | 28234 | 28093 | 27932 | 86.8 |
| JB-S-B1-C53 | 8206 | 3470 | 3394 | 3272 | 3180 | 38.8 |
| JB-S-B1-C54 | 37803 | 32946 | 32833 | 32284 | 32221 | 85.2 |
| JB-S-B1-C55 | 11901 | 8953 | 8903 | 8787 | 8784 | 73.8 |
| JB-S-B1-C56 | 41632 | 36077 | 35863 | 35390 | 35024 | 84.1 |
| JB-S-B1-C57 | 38330 | 33874 | 33737 | 33259 | 33177 | 86.6 |
| JB-S-B1-C58 | 28495 | 25395 | 25274 | 24976 | 24386 | 85.6 |
| JB-S-B1-C59 | 27764 | 24014 | 23825 | 23416 | 23351 | 84.1 |
| JB-S-B1-C60 | 37723 | 30277 | 30175 | 29747 | 29634 | 78.6 |
| JB-S-B1-C61 | 37952 | 33332 | 33209 | 32824 | 32623 | 86 |
| JB-S-B1-C62 | 43101 | 38483 | 38371 | 37893 | 36988 | 85.8 |
| JB-S-B1-C63 | 38172 | 33280 | 33103 | 32817 | 32727 | 85.7 |
| JB-S-B1-C64 | 11340 | 7726 | 7658 | 7481 | 7445 | 65.7 |
| JB-S-B1-C65 | 41512 | 36494 | 36386 | 35980 | 35670 | 85.9 |
| JB-S-B1-C66 | 39094 | 34298 | 34196 | 33882 | 33731 | 86.3 |
| JB-S-B1-C67 | 43203 | 37648 | 37584 | 37250 | 37011 | 85.7 |
| JB-S-B1-DNA-A11 | 45634 | 40530 | 40433 | 39842 | 39735 | 87.1 |
| JB-S-B1-DNA-A3 | 20863 | 18538 | 18492 | 18015 | 18015 | 86.3 |
| JB-S-B1-DNA-F9 | 46370 | 40866 | 40759 | 40109 | 39881 | 86 |
| JB-S-B1-DNA-G2 | 43082 | 37663 | 37405 | 36570 | 36400 | 84.5 |
| JB-S-B1-M01 | 63275 | 55417 | 55180 | 54710 | 54579 | 86.3 |
| JB-S-B1-M02 | 37146 | 30513 | 30444 | 30206 | 30044 | 80.9 |
| JB-S-B1-M03 | 37166 | 33187 | 33055 | 32636 | 32565 | 87.6 |
| JB-S-B1-M04 | 19819 | 17459 | 17413 | 17286 | 17258 | 87.1 |
| JB-S-B1-M05 | 19767 | 17104 | 17051 | 16820 | 16804 | 85 |
| JB-S-B1-M06 | 28774 | 20934 | 20846 | 20689 | 20643 | 71.7 |
| JB-S-B1-M07 | 22654 | 19453 | 19351 | 19154 | 19140 | 84.5 |
| JB-S-B1-M08 | 17488 | 14919 | 14827 | 14405 | 14382 | 82.2 |
| JB-S-B1-M09 | 40043 | 35811 | 35703 | 34853 | 34492 | 86.1 |
| JB-S-B1-M10 | 48976 | 44264 | 44116 | 43457 | 43128 | 88.1 |
| JB-S-B1-M11 | 29419 | 26221 | 26112 | 25813 | 25782 | 87.6 |
| JB-S-B1-M12 | 38334 | 34109 | 34017 | 33517 | 33500 | 87.4 |
| JB-S-B1-M13 | 45142 | 40113 | 39977 | 39602 | 39432 | 87.4 |
| JB-S-B1-M14 | 53649 | 46699 | 46505 | 46056 | 45930 | 85.6 |
| JB-S-B1-M15 | 49837 | 44877 | 44756 | 44317 | 44202 | 88.7 |
| JB-S-B1-M16 | 51055 | 44253 | 44012 | 43439 | 43356 | 84.9 |
| JB-S-B1-M17 | 48243 | 43625 | 43431 | 42992 | 42856 | 88.8 |
| JB-S-B1-M20 | 56434 | 47735 | 47566 | 46651 | 46579 | 82.5 |
| JB-S-B1-M35 | 22728 | 18924 | 18836 | 18618 | 18507 | 81.4 |
| JB-S-B1-M36 | 40198 | 33708 | 33626 | 33302 | 33296 | 82.8 |
| JB-S-B1-M37 | 20287 | 13950 | 13865 | 13635 | 13573 | 66.9 |
| JB-S-B1-M38 | 62375 | 54340 | 54128 | 53593 | 53525 | 85.8 |
| JB-S-B1-M39 | 46991 | 41558 | 41407 | 40972 | 40860 | 87 |
| JB-S-B1-M40 | 49657 | 42954 | 42743 | 42040 | 41858 | 84.3 |
| JB-S-B1-M41 | 51093 | 44643 | 44496 | 43864 | 43821 | 85.8 |
| JB-S-B1-M42 | 44093 | 39452 | 39362 | 39142 | 39076 | 88.6 |
| JB-S-B1-M43 | 40317 | 35204 | 35075 | 34694 | 34634 | 85.9 |
| JB-S-B1-M44 | 34048 | 30208 | 30126 | 29570 | 29490 | 86.6 |
| JB-S-B1-M45 | 31600 | 27361 | 27255 | 26881 | 26856 | 85 |
| JB-S-B1-M46 | 27931 | 24685 | 24627 | 24425 | 24388 | 87.3 |
| JB-S-B1-M47 | 36861 | 32051 | 31989 | 31809 | 31780 | 86.2 |
| JB-S-B1-M65 | 34140 | 30278 | 30200 | 29844 | 29844 | 87.4 |
| JB-S-B1-M66 | 44363 | 39798 | 39720 | 39340 | 39303 | 88.6 |
| JB-S-B1-M71 | 31936 | 28336 | 28203 | 27947 | 27935 | 87.5 |
| JB-S-B1-M72 | 49083 | 44104 | 44029 | 43605 | 43515 | 88.7 |
| JB-S-B1-M73 | 55546 | 47602 | 47444 | 46725 | 46625 | 83.9 |
| JB-S-B1-M74 | 51866 | 45636 | 45444 | 44820 | 44665 | 86.1 |
| JB-S-B1-M75 | 41099 | 34883 | 34745 | 34438 | 34349 | 83.6 |
| JB-S-B1-PCR1-C4 | 30 | 1 | 1 | 1 | 1 | 3.3 |
| JB-S-B1-PCR2-A4 | 3 | 2 | 1 | 0 | 0 | 0 |
| JB-S-B2-C09 | 61953 | 54236 | 54111 | 53228 | 51618 | 83.3 |
| JB-S-B2-C10 | 61865 | 54669 | 54416 | 53972 | 53646 | 86.7 |
| JB-S-B2-C11 | 42448 | 36904 | 36817 | 36367 | 36035 | 84.9 |
| JB-S-B2-C12 | 50219 | 44709 | 44550 | 44016 | 43380 | 86.4 |
| JB-S-B2-C13 | 12449 | 9148 | 9060 | 8858 | 8740 | 70.2 |
| JB-S-B2-C14 | 26948 | 23541 | 23471 | 23310 | 23271 | 86.4 |
| JB-S-B2-C15 | 35783 | 29883 | 29763 | 29558 | 29298 | 81.9 |
| JB-S-B2-C16 | 51919 | 45602 | 45451 | 44991 | 44604 | 85.9 |
| JB-S-B2-C25 | 22100 | 19012 | 18937 | 18763 | 18738 | 84.8 |
| JB-S-B2-C27 | 64928 | 57329 | 57186 | 56604 | 55292 | 85.2 |
| JB-S-B2-C28 | 9563 | 8532 | 8470 | 8206 | 8188 | 85.6 |
| JB-S-B2-C29 | 33149 | 28587 | 28472 | 28310 | 28163 | 85 |
| JB-S-B2-C30 | 31361 | 27460 | 27404 | 27237 | 27225 | 86.8 |
| JB-S-B2-C31 | 25554 | 22490 | 22443 | 22292 | 22287 | 87.2 |
| JB-S-B2-C32 | 36462 | 32323 | 32241 | 31967 | 31938 | 87.6 |
| JB-S-B2-C41 | 45883 | 39159 | 39051 | 38786 | 38529 | 84 |
| JB-S-B2-C42 | 49746 | 43846 | 43685 | 43251 | 42902 | 86.2 |
| JB-S-B2-C43 | 18067 | 15835 | 15773 | 15614 | 15614 | 86.4 |
| JB-S-B2-C44 | 23996 | 21407 | 21319 | 21083 | 20872 | 87 |
| JB-S-B2-C45 | 30954 | 27051 | 26991 | 26396 | 26396 | 85.3 |
| JB-S-B2-C46 | 20903 | 18112 | 18030 | 17843 | 17712 | 84.7 |
| JB-S-B2-C47 | 44465 | 39866 | 39741 | 39376 | 38730 | 87.1 |
| JB-S-B2-C48 | 51513 | 45494 | 45330 | 44945 | 44716 | 86.8 |
| JB-S-B2-C68 | 39212 | 33526 | 33399 | 33131 | 33015 | 84.2 |
| JB-S-B2-C69 | 45819 | 41394 | 41242 | 40825 | 40578 | 88.6 |
| JB-S-B2-C70 | 23064 | 20409 | 20343 | 20037 | 20002 | 86.7 |
| JB-S-B2-C71 | 38408 | 33980 | 33852 | 33591 | 33396 | 87 |
| JB-S-B2-C72 | 41918 | 36233 | 36031 | 35615 | 35535 | 84.8 |
| JB-S-B2-C73 | 30628 | 27544 | 27461 | 27138 | 27109 | 88.5 |
| JB-S-B2-C74 | 41496 | 36239 | 36157 | 35798 | 35607 | 85.8 |
| JB-S-B2-C75 | 15386 | 13412 | 13361 | 13190 | 13181 | 85.7 |
| JB-S-B2-DNA-G3 | 35964 | 33089 | 33029 | 32611 | 32611 | 90.7 |
| JB-S-B2-DNA-G5 | 13388 | 11996 | 11915 | 11621 | 11621 | 86.8 |
| JB-S-B2-M18 | 58020 | 52010 | 51846 | 51232 | 51016 | 87.9 |
| JB-S-B2-M19 | 63359 | 57247 | 57079 | 56444 | 56094 | 88.5 |
| JB-S-B2-M21 | 41882 | 37558 | 37475 | 37102 | 37062 | 88.5 |
| JB-S-B2-M22 | 57644 | 51981 | 51876 | 49835 | 49671 | 86.2 |
| JB-S-B2-M23 | 55629 | 50294 | 50024 | 49215 | 49033 | 88.1 |
| JB-S-B2-M24 | 38812 | 34966 | 34825 | 34467 | 34297 | 88.4 |
| JB-S-B2-M25 | 44220 | 39707 | 39576 | 39295 | 38856 | 87.9 |
| JB-S-B2-M26 | 63743 | 55832 | 55647 | 54642 | 54320 | 85.2 |
| JB-S-B2-M27 | 47516 | 42443 | 42318 | 41844 | 41618 | 87.6 |
| JB-S-B2-M28 | 32410 | 29231 | 29139 | 28561 | 28515 | 88 |
| JB-S-B2-M29 | 16030 | 14704 | 14636 | 14352 | 14348 | 89.5 |
| JB-S-B2-M30 | 27598 | 23133 | 22994 | 22536 | 22536 | 81.7 |
| JB-S-B2-M31 | 40703 | 35850 | 35753 | 35438 | 35366 | 86.9 |
| JB-S-B2-M32 | 21326 | 19218 | 19106 | 18972 | 18966 | 88.9 |
| JB-S-B2-M33 | 42721 | 37583 | 37519 | 36721 | 36689 | 85.9 |
| JB-S-B2-M34 | 36622 | 31826 | 31749 | 31503 | 31468 | 85.9 |
| JB-S-B2-M49 | 30902 | 26942 | 26817 | 26495 | 26471 | 85.7 |
| JB-S-B2-M50 | 32569 | 29206 | 29091 | 28736 | 28469 | 87.4 |
| JB-S-B2-M51 | 8401 | 7547 | 7488 | 7396 | 7396 | 88 |
| JB-S-B2-M52 | 40477 | 35942 | 35866 | 35565 | 35519 | 87.8 |
| JB-S-B2-M53 | 26318 | 22837 | 22711 | 22230 | 22206 | 84.4 |
| JB-S-B2-M54 | 36228 | 32694 | 32615 | 32049 | 32013 | 88.4 |
| JB-S-B2-M55 | 30973 | 25861 | 25789 | 25593 | 25518 | 82.4 |
| JB-S-B2-M56 | 24619 | 22136 | 22031 | 21565 | 21366 | 86.8 |
| JB-S-B2-PCR1-H5 | 221 | 182 | 181 | 181 | 181 | 81.9 |
| JB-W-B1-10 | 42176 | 37306 | 37162 | 36619 | 36081 | 85.5 |
| JB-W-B1-11 | 29859 | 26648 | 26570 | 26337 | 26094 | 87.4 |
| JB-W-B1-12 | 50145 | 44003 | 43917 | 43574 | 43118 | 86 |
| JB-W-B1-13 | 52613 | 46028 | 45905 | 45475 | 45380 | 86.3 |
| JB-W-B1-14 | 39848 | 34393 | 34299 | 34053 | 33559 | 84.2 |
| JB-W-B1-15 | 52803 | 45893 | 45756 | 45400 | 45127 | 85.5 |
| JB-W-B1-16 | 41908 | 36061 | 35895 | 35699 | 35619 | 85 |
| JB-W-B1-17 | 18986 | 16615 | 16585 | 16547 | 16547 | 87.2 |
| JB-W-B1-18 | 8314 | 7238 | 7227 | 7122 | 7122 | 85.7 |
| JB-W-B1-19 | 16848 | 14630 | 14606 | 14518 | 14508 | 86.1 |
| JB-W-B1-20 | 39776 | 33760 | 33635 | 33288 | 33168 | 83.4 |
| JB-W-B1-21 | 70827 | 53143 | 52971 | 52567 | 52470 | 74.1 |
| JB-W-B1-22 | 26331 | 22616 | 22566 | 22253 | 22243 | 84.5 |
| JB-W-B1-23 | 18931 | 15495 | 15468 | 15422 | 15422 | 81.5 |
| JB-W-B1-24 | 34995 | 30757 | 30669 | 30245 | 30122 | 86.1 |
| JB-W-B1-25 | 57461 | 50250 | 50177 | 49951 | 49544 | 86.2 |
| JB-W-B1-26 | 47365 | 41733 | 41667 | 41550 | 41459 | 87.5 |
| JB-W-B1-27 | 44426 | 38424 | 38345 | 38135 | 38000 | 85.5 |
| JB-W-B1-28 | 31080 | 27239 | 27201 | 26997 | 26900 | 86.6 |
| JB-W-B1-29 | 20170 | 17250 | 17219 | 17138 | 17138 | 85 |
| JB-W-B1-30 | 45232 | 40143 | 40053 | 39716 | 39589 | 87.5 |
| JB-W-B1-31 | 30840 | 26059 | 25998 | 25882 | 25863 | 83.9 |
| JB-W-B1-32 | 66714 | 59308 | 59080 | 58379 | 58099 | 87.1 |
| JB-W-B1-33 | 60977 | 52571 | 52412 | 52210 | 51944 | 85.2 |
| JB-W-B1-34 | 57508 | 48328 | 48179 | 47488 | 47284 | 82.2 |
| JB-W-B1-35 | 75317 | 66649 | 66376 | 65654 | 65233 | 86.6 |
| JB-W-B1-36 | 28676 | 23796 | 23681 | 23521 | 23444 | 81.8 |
| JB-W-B1-37 | 56547 | 48702 | 48521 | 48220 | 48177 | 85.2 |
| JB-W-B1-38 | 46290 | 34323 | 34250 | 33996 | 33974 | 73.4 |
| JB-W-B1-39 | 67505 | 59183 | 59020 | 58363 | 56930 | 84.3 |
| JB-W-B1-40 | 115637 | 103558 | 103317 | 102350 | 99731 | 86.2 |
| JB-W-B1-41 | 4095 | 3597 | 3580 | 3554 | 3554 | 86.8 |
| JB-W-B1-42 | 34115 | 29665 | 29549 | 29157 | 29120 | 85.4 |
| JB-W-B1-43 | 22874 | 20309 | 20265 | 20161 | 20154 | 88.1 |
| JB-W-B1-44 | 19884 | 17138 | 17119 | 17048 | 17048 | 85.7 |
| JB-W-B1-45 | 56367 | 49210 | 49056 | 48779 | 48637 | 86.3 |
| JB-W-B1-46 | 33312 | 28533 | 28465 | 28280 | 28264 | 84.8 |
| JB-W-B1-47 | 3235 | 1324 | 1245 | 1106 | 1106 | 34.2 |
| JB-W-B1-48 | 19707 | 17547 | 17528 | 17487 | 16845 | 85.5 |
| JB-W-B1-49 | 24352 | 15280 | 15254 | 15132 | 15132 | 62.1 |
| JB-W-B1-50 | 11785 | 10147 | 10121 | 10024 | 10024 | 85.1 |
| JB-W-B1-51 | 21033 | 18351 | 18329 | 18249 | 18249 | 86.8 |
| JB-W-B1-52 | 5645 | 4614 | 4601 | 4587 | 4587 | 81.3 |
| JB-W-B1-53 | 32871 | 27585 | 27534 | 27399 | 27385 | 83.3 |
| JB-W-B1-54 | 18885 | 16015 | 15910 | 15811 | 15786 | 83.6 |
| JB-W-B1-55 | 50694 | 44491 | 44425 | 44138 | 44045 | 86.9 |
| JB-W-B1-56 | 54121 | 47488 | 47381 | 47015 | 46969 | 86.8 |
| JB-W-B1-57 | 50635 | 44399 | 44319 | 44046 | 44046 | 87 |
| JB-W-B1-58 | 61585 | 54052 | 53892 | 53490 | 53325 | 86.6 |
| JB-W-B1-59 | 58315 | 50811 | 50719 | 50500 | 50148 | 86 |
| JB-W-B1-60 | 49797 | 43490 | 43421 | 43303 | 43281 | 86.9 |
| JB-W-B1-61 | 61153 | 50542 | 50430 | 49879 | 49807 | 81.4 |
| JB-W-B1-62 | 72653 | 63326 | 63171 | 62654 | 62499 | 86 |
| JB-W-B1-63 | 36265 | 31813 | 31708 | 31585 | 31580 | 87.1 |
| JB-W-B1-64 | 35255 | 31481 | 31408 | 31198 | 30944 | 87.8 |
| JB-W-B1-65 | 29144 | 25360 | 25324 | 25147 | 25141 | 86.3 |
| JB-W-B1-66 | 26009 | 22442 | 22403 | 22195 | 22169 | 85.2 |
| JB-W-B1-67 | 12044 | 9705 | 9668 | 9607 | 9607 | 79.8 |
| JB-W-B1-68 | 28689 | 25112 | 25081 | 24837 | 24827 | 86.5 |
| JB-W-B1-69 | 32414 | 27871 | 27805 | 27513 | 27505 | 84.9 |
| JB-W-B1-70 | 16924 | 14222 | 14194 | 13674 | 13674 | 80.8 |
| JB-W-B1-71 | 48591 | 42221 | 41729 | 40988 | 40967 | 84.3 |
| JB-W-B1-72 | 56504 | 48810 | 48739 | 48295 | 47208 | 83.5 |
| JB-W-B1-73 | 25185 | 21627 | 21624 | 21216 | 21215 | 84.2 |
| JB-W-B1-74 | 58225 | 52657 | 52569 | 52070 | 50709 | 87.1 |
| JB-W-B1-75 | 41137 | 37708 | 37639 | 37306 | 36052 | 87.6 |
| JB-W-B1-76 | 25578 | 22546 | 22504 | 22445 | 22427 | 87.7 |
| JB-W-B1-77 | 21678 | 18836 | 18799 | 18631 | 18616 | 85.9 |
| JB-W-B1-78 | 52108 | 46308 | 46192 | 45758 | 45673 | 87.7 |
| JB-W-B1-79 | 59670 | 50052 | 49721 | 49499 | 49402 | 82.8 |
| JB-W-B1-80 | 36611 | 31568 | 31429 | 30891 | 30850 | 84.3 |
| JB-W-B1-81 | 54527 | 47520 | 47239 | 46901 | 46889 | 86 |
| JB-W-B1-82 | 44409 | 39128 | 38875 | 38364 | 38360 | 86.4 |
| JB-W-B1-83 | 40460 | 35381 | 35297 | 34896 | 34880 | 86.2 |
| JB-W-B1-84 | 14067 | 11412 | 11401 | 11201 | 11201 | 79.6 |
| JB-W-B1-85 | 20249 | 16710 | 16681 | 16480 | 16480 | 81.4 |
| JB-W-B1-9 | 61720 | 54300 | 54099 | 53537 | 53077 | 86 |
| JB-W-B1-DNA-A4 | 55951 | 49007 | 48859 | 48449 | 48222 | 86.2 |
| JB-W-B1-DNA-A5 | 69746 | 60806 | 60699 | 59830 | 59650 | 85.5 |
| JB-W-B1-DNA-A9 | 26552 | 23401 | 23326 | 23134 | 23134 | 87.1 |
| JB-W-B1-PCR1-F6 | 159 | 144 | 144 | 144 | 144 | 90.6 |
| JB-W-B1-PCR1-H6 | 46 | 34 | 32 | 0 | 0 | 0 |
| JB-W-B2-100 | 36421 | 28603 | 28549 | 28427 | 28427 | 78.1 |
| JB-W-B2-101 | 28956 | 25724 | 25657 | 25458 | 25440 | 87.9 |
| JB-W-B2-102 | 22381 | 19805 | 19761 | 19505 | 19485 | 87.1 |
| JB-W-B2-103 | 21491 | 17059 | 16972 | 16779 | 16769 | 78 |
| JB-W-B2-104 | 36522 | 31480 | 31400 | 31095 | 31028 | 85 |
| JB-W-B2-105 | 46403 | 40269 | 40169 | 39743 | 39115 | 84.3 |
| JB-W-B2-106 | 40193 | 33197 | 33088 | 32683 | 32564 | 81 |
| JB-W-B2-107 | 14704 | 12649 | 12591 | 12417 | 12214 | 83.1 |
| JB-W-B2-108 | 35892 | 30662 | 30566 | 30460 | 30281 | 84.4 |
| JB-W-B2-109 | 77341 | 68748 | 68567 | 68053 | 67694 | 87.5 |
| JB-W-B2-110 | 52366 | 45336 | 45286 | 45203 | 45203 | 86.3 |
| JB-W-B2-111 | 53930 | 43863 | 43812 | 43725 | 43670 | 81 |
| JB-W-B2-112 | 38091 | 34042 | 33967 | 33883 | 33878 | 88.9 |
| JB-W-B2-113 | 31423 | 27348 | 27312 | 27162 | 27073 | 86.2 |
| JB-W-B2-114 | 56533 | 46308 | 46232 | 46068 | 46041 | 81.4 |
| JB-W-B2-115 | 49476 | 43993 | 43906 | 43708 | 43485 | 87.9 |
| JB-W-B2-116 | 30335 | 25427 | 25380 | 25293 | 25293 | 83.4 |
| JB-W-B2-117 | 52364 | 46184 | 46094 | 45828 | 45801 | 87.5 |
| JB-W-B2-118 | 49765 | 42854 | 42792 | 42641 | 42567 | 85.5 |
| JB-W-B2-119 | 37085 | 30966 | 30908 | 30731 | 30718 | 82.8 |
| JB-W-B2-120 | 41875 | 37705 | 37587 | 37342 | 37245 | 88.9 |
| JB-W-B2-121 | 55392 | 50342 | 50259 | 49869 | 49819 | 89.9 |
| JB-W-B2-122 | 55716 | 48623 | 48543 | 48368 | 48303 | 86.7 |
| JB-W-B2-123 | 76466 | 68304 | 68209 | 67817 | 67584 | 88.4 |
| JB-W-B2-124 | 62505 | 54326 | 54214 | 53795 | 53756 | 86 |
| JB-W-B2-125 | 46107 | 41380 | 41245 | 40829 | 40799 | 88.5 |
| JB-W-B2-126 | 38070 | 33514 | 33476 | 33344 | 33344 | 87.6 |
| JB-W-B2-127 | 46541 | 41045 | 40933 | 40550 | 40462 | 86.9 |
| JB-W-B2-128 | 38185 | 33962 | 33858 | 33670 | 33660 | 88.1 |
| JB-W-B2-129 | 14939 | 12635 | 12600 | 12465 | 12465 | 83.4 |
| JB-W-B2-130 | 42964 | 36293 | 36242 | 35721 | 35721 | 83.1 |
| JB-W-B2-131 | 17589 | 15579 | 15561 | 15453 | 15453 | 87.9 |
| JB-W-B2-132 | 50272 | 43173 | 43003 | 42204 | 42056 | 83.7 |
| JB-W-B2-133 | 55525 | 49094 | 49029 | 48766 | 48748 | 87.8 |
| JB-W-B2-134 | 33615 | 29943 | 29872 | 29788 | 29788 | 88.6 |
| JB-W-B2-135 | 43273 | 35521 | 35464 | 35407 | 35407 | 81.8 |
| JB-W-B2-136 | 28101 | 25301 | 25231 | 24387 | 24387 | 86.8 |
| JB-W-B2-137 | 69402 | 61789 | 61700 | 61308 | 60803 | 87.6 |
| JB-W-B2-138 | 39258 | 34407 | 34397 | 33781 | 33781 | 86 |
| JB-W-B2-139 | 53392 | 46749 | 46650 | 46441 | 46419 | 86.9 |
| JB-W-B2-140 | 41621 | 35677 | 35615 | 34730 | 34725 | 83.4 |
| JB-W-B2-141 | 42464 | 36258 | 36208 | 36077 | 36077 | 85 |
| JB-W-B2-142 | 23000 | 19835 | 19712 | 19497 | 19497 | 84.8 |
| JB-W-B2-143 | 23440 | 14857 | 14835 | 14539 | 14536 | 62 |
| JB-W-B2-144 | 17555 | 15587 | 15532 | 15355 | 15355 | 87.5 |
| JB-W-B2-145 | 7146 | 6347 | 6334 | 6223 | 6223 | 87.1 |
| JB-W-B2-146 | 34379 | 29435 | 29364 | 29114 | 29083 | 84.6 |
| JB-W-B2-147 | 25299 | 22368 | 22331 | 22285 | 22277 | 88.1 |
| JB-W-B2-148 | 56471 | 50955 | 50859 | 50361 | 50304 | 89.1 |
| JB-W-B2-149 | 78123 | 68807 | 68659 | 68192 | 67592 | 86.5 |
| JB-W-B2-150 | 49609 | 36285 | 36194 | 36015 | 35946 | 72.5 |
| JB-W-B2-151 | 85611 | 75724 | 75592 | 74945 | 74362 | 86.9 |
| JB-W-B2-152 | 57377 | 51823 | 51698 | 51216 | 50990 | 88.9 |
| JB-W-B2-153 | 43052 | 36423 | 36381 | 36182 | 36182 | 84 |
| JB-W-B2-154 | 43546 | 37595 | 37505 | 37074 | 37074 | 85.1 |
| JB-W-B2-155 | 16235 | 13021 | 12997 | 12880 | 12880 | 79.3 |
| JB-W-B2-156 | 45632 | 40558 | 40393 | 40050 | 40022 | 87.7 |
| JB-W-B2-157 | 45711 | 40234 | 40181 | 39771 | 39771 | 87 |
| JB-W-B2-158 | 34021 | 28823 | 28775 | 28604 | 28576 | 84 |
| JB-W-B2-159 | 28512 | 25831 | 25787 | 25663 | 25428 | 89.2 |
| JB-W-B2-160 | 52099 | 46713 | 46528 | 46243 | 46176 | 88.6 |
| JB-W-B2-86 | 2654 | 2319 | 2317 | 2314 | 2314 | 87.2 |
| JB-W-B2-87 | 14297 | 12246 | 12233 | 12122 | 12122 | 84.8 |
| JB-W-B2-88 | 35014 | 26494 | 26458 | 26374 | 26374 | 75.3 |
| JB-W-B2-89 | 40722 | 36059 | 35969 | 35678 | 35591 | 87.4 |
| JB-W-B2-90 | 29443 | 26044 | 25980 | 25873 | 25873 | 87.9 |
| JB-W-B2-91 | 28481 | 24053 | 24012 | 23773 | 23711 | 83.3 |
| JB-W-B2-92 | 32729 | 28436 | 28391 | 28242 | 28242 | 86.3 |
| JB-W-B2-93 | 21028 | 15261 | 15203 | 15145 | 15145 | 72 |
| JB-W-B2-94 | 46231 | 41022 | 40922 | 40592 | 40592 | 87.8 |
| JB-W-B2-95 | 40096 | 34200 | 34056 | 33310 | 33310 | 83.1 |
| JB-W-B2-96 | 41622 | 33839 | 33763 | 33325 | 33242 | 79.9 |
| JB-W-B2-97 | 51262 | 45216 | 45137 | 44997 | 44965 | 87.7 |
| JB-W-B2-98 | 1463 | 1252 | 1248 | 1244 | 1244 | 85 |
| JB-W-B2-99 | 31682 | 26500 | 26466 | 26374 | 26374 | 83.2 |
| JB-W-B2-DNA-A1 | 37127 | 31267 | 31201 | 30862 | 30862 | 83.1 |
| JB-W-B2-DNA-A8 | 9669 | 7730 | 7713 | 7453 | 7453 | 77.1 |
| JB-W-B2-DNA-C7 | 69377 | 59630 | 59532 | 59220 | 59129 | 85.2 |
| JB-W-B2-PCR1-C12 | 1487 | 1308 | 1306 | 1304 | 1304 | 87.7 |
| JB-W-B2-PCR1-F6 | 871 | 754 | 754 | 753 | 753 | 86.5 |
| JB-W-B2-PCR1-H6 | 429 | 367 | 367 | 367 | 367 | 85.5 |
| JB_W_B1_DNA_NC_A6 | 16074 | 15753 | 15751 | 15747 | 15747 | 98 |
| JB_W_B1_DNA_NC_B6 | 1925 | 1891 | 1829 | 1740 | 1740 | 90.4 |
| JB_W_B1_DNA_NC_G12 | 18586 | 18250 | 18247 | 18242 | 18238 | 98.1 |
| JB_W_B1_DNA_NC_H12 | 903 | 893 | 877 | 877 | 877 | 97.1 |
| JB_W_B1_M033 | 14548 | 14029 | 14028 | 13980 | 13980 | 96.1 |
| JB_W_B1_M034 | 10019 | 9856 | 9853 | 9853 | 9853 | 98.3 |
| JB_W_B1_M035 | 10078 | 9902 | 9900 | 9745 | 9745 | 96.7 |
| JB_W_B1_M036 | 8694 | 8531 | 8529 | 8472 | 8472 | 97.4 |
| JB_W_B1_M037 | 5769 | 5566 | 5463 | 5439 | 5424 | 94 |
| JB_W_B1_M038 | 13275 | 12934 | 12930 | 12866 | 12866 | 96.9 |
| JB_W_B1_M039 | 12589 | 12357 | 12356 | 12350 | 12350 | 98.1 |
| JB_W_B1_M040 | 7332 | 7197 | 7194 | 7188 | 7188 | 98 |
| JB_W_B1_M049 | 27102 | 26591 | 26559 | 26539 | 26413 | 97.5 |
| JB_W_B1_M050 | 4755 | 4671 | 4669 | 4632 | 4632 | 97.4 |
| JB_W_B1_M051 | 14291 | 14060 | 14058 | 14055 | 14055 | 98.3 |
| JB_W_B1_M052 | 19819 | 19412 | 19411 | 19408 | 19408 | 97.9 |
| JB_W_B1_M054 | 18260 | 17307 | 17306 | 17305 | 17305 | 94.8 |
| JB_W_B1_M055 | 13882 | 13635 | 13635 | 13632 | 13632 | 98.2 |
| JB_W_B1_M056 | 11780 | 11386 | 11382 | 11346 | 11346 | 96.3 |
| JB_W_B1_M065 | 14948 | 12619 | 12586 | 12583 | 12583 | 84.2 |
| JB_W_B1_M066 | 9748 | 9565 | 9485 | 9417 | 9417 | 96.6 |
| JB_W_B1_M067 | 11772 | 11560 | 11560 | 11532 | 11532 | 98 |
| JB_W_B1_M068 | 18834 | 18485 | 18483 | 18473 | 18473 | 98.1 |
| JB_W_B1_M069 | 13381 | 13107 | 13103 | 13057 | 13057 | 97.6 |
| JB_W_B1_M070 | 5500 | 5358 | 5358 | 5358 | 5358 | 97.4 |
| JB_W_B1_M071 | 4785 | 4695 | 4694 | 4694 | 4694 | 98.1 |
| JB_W_B1_M072 | 5069 | 4965 | 4965 | 4963 | 4963 | 97.9 |
| JB_W_B1_M073 | 20814 | 20249 | 20236 | 19899 | 19899 | 95.6 |
| JB_W_B1_M074 | 18366 | 18015 | 18009 | 17947 | 17947 | 97.7 |
| JB_W_B1_M075 | 6228 | 6116 | 6114 | 6110 | 6110 | 98.1 |
| JB_W_B1_M076 | 15890 | 15610 | 15608 | 15533 | 15533 | 97.8 |
| JB_W_B1_M077 | 16761 | 16455 | 16449 | 16434 | 16423 | 98 |
| JB_W_B1_M078 | 15840 | 15538 | 15529 | 15527 | 15527 | 98 |
| JB_W_B1_M079 | 11580 | 11371 | 11362 | 11323 | 11318 | 97.7 |
| JB_W_B1_M080 | 26199 | 25757 | 25567 | 25362 | 25327 | 96.7 |
| JB_W_B1_M081 | 17905 | 17548 | 17536 | 17514 | 17496 | 97.7 |
| JB_W_B1_M082 | 31660 | 31078 | 31075 | 31049 | 31029 | 98 |
| JB_W_B1_M083 | 7768 | 7628 | 7626 | 7621 | 7621 | 98.1 |
| JB_W_B1_M084 | 18930 | 18542 | 18505 | 18436 | 18403 | 97.2 |
| JB_W_B1_M085 | 15245 | 15041 | 15024 | 14958 | 14913 | 97.8 |
| JB_W_B1_M086 | 11631 | 11394 | 11380 | 11357 | 11345 | 97.5 |
| JB_W_B1_M087 | 17996 | 17663 | 17658 | 17650 | 17645 | 98 |
| JB_W_B1_M088 | 17796 | 17436 | 17433 | 17424 | 17424 | 97.9 |
| JB_W_B1_M089 | 19980 | 17170 | 17158 | 17153 | 17153 | 85.9 |
| JB_W_B1_M090 | 17994 | 17626 | 17613 | 17592 | 17582 | 97.7 |
| JB_W_B1_M091 | 17965 | 17574 | 17559 | 17450 | 17266 | 96.1 |
| JB_W_B1_M092 | 38569 | 37940 | 37916 | 37766 | 37440 | 97.1 |
| JB_W_B1_M093 | 4768 | 4539 | 4533 | 4529 | 4529 | 95 |
| JB_W_B1_M094 | 22669 | 22169 | 22155 | 22077 | 21970 | 96.9 |
| JB_W_B1_M095 | 5306 | 5115 | 5114 | 5108 | 5108 | 96.3 |
| JB_W_B1_M113 | 14908 | 14470 | 14462 | 14324 | 14302 | 95.9 |
| JB_W_B1_M114 | 12529 | 12278 | 12262 | 12243 | 12243 | 97.7 |
| JB_W_B1_M115 | 6353 | 6218 | 6211 | 6163 | 6163 | 97 |
| JB_W_B1_M116 | 12933 | 12725 | 12716 | 12676 | 12625 | 97.6 |
| JB_W_B1_M117 | 4084 | 4010 | 4010 | 4008 | 4008 | 98.1 |
| JB_W_B1_M118 | 5439 | 5341 | 5334 | 5322 | 5322 | 97.8 |
| JB_W_B1_M119 | 2653 | 2584 | 2583 | 2578 | 2578 | 97.2 |
| JB_W_B1_M120 | 8417 | 8255 | 8255 | 8253 | 8253 | 98.1 |
| JB_W_B1_M121 | 8925 | 8762 | 8750 | 8735 | 8735 | 97.9 |
| JB_W_B1_M122 | 17303 | 17019 | 16951 | 16942 | 16940 | 97.9 |
| JB_W_B1_M123 | 16142 | 15858 | 15858 | 15823 | 15823 | 98 |
| JB_W_B1_M124 | 15801 | 15530 | 15521 | 15492 | 15481 | 98 |
| JB_W_B1_M125 | 20122 | 19795 | 19791 | 19788 | 19788 | 98.3 |
| JB_W_B1_M126 | 13383 | 13122 | 13075 | 12980 | 12980 | 97 |
| JB_W_B1_M127 | 9620 | 9424 | 9411 | 9409 | 9409 | 97.8 |
| JB_W_B1_M128 | 14701 | 14442 | 14435 | 14329 | 14316 | 97.4 |
| JB_W_B1_M129 | 17888 | 17487 | 17473 | 17413 | 17371 | 97.1 |
| JB_W_B1_M130 | 3721 | 3658 | 3651 | 3644 | 3644 | 97.9 |
| JB_W_B1_M131 | 14048 | 13799 | 13798 | 13793 | 13793 | 98.2 |
| JB_W_B1_M132 | 8507 | 8368 | 8367 | 8367 | 8367 | 98.4 |
| JB_W_B1_M133 | 10897 | 10729 | 10722 | 10713 | 10713 | 98.3 |
| JB_W_B1_M134 | 9986 | 9761 | 9750 | 9745 | 9745 | 97.6 |
| JB_W_B1_M135 | 11510 | 11273 | 11267 | 11236 | 11224 | 97.5 |
| JB_W_B1_M136 | 1256 | 1196 | 1196 | 1196 | 1196 | 95.2 |
| JB_W_B1_M145 | 20837 | 20354 | 20297 | 20224 | 20224 | 97.1 |
| JB_W_B1_M146 | 8150 | 7984 | 7984 | 7953 | 7953 | 97.6 |
| JB_W_B1_M147 | 13709 | 13424 | 13423 | 13406 | 13406 | 97.8 |
| JB_W_B1_M148 | 5793 | 5675 | 5675 | 5675 | 5675 | 98 |
| JB_W_B1_M149 | 16983 | 16648 | 16646 | 16590 | 16590 | 97.7 |
| JB_W_B1_M150 | 9787 | 9592 | 9586 | 9576 | 9576 | 97.8 |
| JB_W_B1_M151 | 10100 | 9931 | 9928 | 9926 | 9926 | 98.3 |
| JB_W_B1_M152 | 3808 | 3734 | 3730 | 3725 | 3725 | 97.8 |
| JB_W_B1_PCR1_D6 | 343 | 336 | 336 | 336 | 336 | 98 |
| JB_W_B2_DNA_NC_A6 | 1622 | 1604 | 1602 | 1555 | 1555 | 95.9 |
| JB_W_B2_DNA_NC_B6 | 3149 | 3093 | 3092 | 3092 | 3092 | 98.2 |
| JB_W_B2_DNA_NC_G12 | 1300 | 1269 | 1269 | 1269 | 1269 | 97.6 |
| JB_W_B2_DNA_NC_H12 | 13317 | 13072 | 13065 | 13044 | 13044 | 97.9 |
| JB_W_B2_M001 | 9654 | 9448 | 9434 | 9401 | 9296 | 96.3 |
| JB_W_B2_M002 | 1281 | 1236 | 1232 | 1232 | 1232 | 96.2 |
| JB_W_B2_M003 | 14924 | 14625 | 14594 | 14522 | 14422 | 96.6 |
| JB_W_B2_M004 | 3911 | 3820 | 3819 | 3817 | 3817 | 97.6 |
| JB_W_B2_M005 | 17331 | 16918 | 16904 | 16881 | 16873 | 97.4 |
| JB_W_B2_M006 | 11761 | 11450 | 11447 | 11422 | 11422 | 97.1 |
| JB_W_B2_M009 | 12018 | 11792 | 11792 | 11790 | 11790 | 98.1 |
| JB_W_B2_M010 | 13447 | 13170 | 13154 | 13043 | 12979 | 96.5 |
| JB_W_B2_M011 | 6344 | 6195 | 6128 | 6037 | 6037 | 95.2 |
| JB_W_B2_M012 | 1387 | 1353 | 1351 | 1350 | 1350 | 97.3 |
| JB_W_B2_M013 | 19495 | 19156 | 19144 | 19138 | 19136 | 98.2 |
| JB_W_B2_M014 | 6117 | 5991 | 5991 | 5983 | 5983 | 97.8 |
| JB_W_B2_M015 | 995 | 962 | 962 | 962 | 962 | 96.7 |
| JB_W_B2_M016 | 4309 | 4227 | 4227 | 4227 | 4227 | 98.1 |
| JB_W_B2_M017 | 22610 | 22190 | 22176 | 21981 | 21981 | 97.2 |
| JB_W_B2_M018 | 37853 | 37266 | 37251 | 36792 | 33390 | 88.2 |
| JB_W_B2_M019 | 32706 | 32028 | 31965 | 31766 | 31440 | 96.1 |
| JB_W_B2_M020 | 28148 | 27550 | 27548 | 27499 | 27493 | 97.7 |
| JB_W_B2_M021 | 22418 | 21706 | 21689 | 21589 | 21505 | 95.9 |
| JB_W_B2_M022 | 7412 | 7164 | 7110 | 7077 | 7077 | 95.5 |
| JB_W_B2_M023 | 32764 | 31676 | 31659 | 31205 | 30989 | 94.6 |
| JB_W_B2_M024 | 34851 | 34170 | 34004 | 33548 | 33424 | 95.9 |
| JB_W_B2_M025 | 4950 | 4858 | 4856 | 4842 | 4842 | 97.8 |
| JB_W_B2_M026 | 4591 | 4456 | 4456 | 4450 | 4450 | 96.9 |
| JB_W_B2_M027 | 7720 | 7591 | 7584 | 7559 | 7528 | 97.5 |
| JB_W_B2_M028 | 992 | 961 | 956 | 955 | 955 | 96.3 |
| JB_W_B2_M029 | 16137 | 15831 | 15828 | 15818 | 15736 | 97.5 |
| JB_W_B2_M030 | 1039 | 1019 | 1018 | 1012 | 1012 | 97.4 |
| JB_W_B2_M031 | 480 | 472 | 467 | 467 | 467 | 97.3 |
| JB_W_B2_M032 | 5220 | 5060 | 5058 | 5052 | 5052 | 96.8 |
| JB_W_B2_M041 | 984 | 952 | 949 | 949 | 949 | 96.4 |
| JB_W_B2_M042 | 10356 | 10155 | 10141 | 10107 | 10025 | 96.8 |
| JB_W_B2_M043 | 6165 | 6045 | 6042 | 6034 | 6034 | 97.9 |
| JB_W_B2_M044 | 5741 | 5624 | 5622 | 5622 | 5622 | 97.9 |
| JB_W_B2_M046 | 7939 | 7763 | 7743 | 7704 | 7704 | 97 |
| JB_W_B2_M047 | 8487 | 8322 | 8322 | 8316 | 8316 | 98 |
| JB_W_B2_M048 | 13660 | 13348 | 13346 | 13253 | 13253 | 97 |
| JB_W_B2_M057 | 1872 | 1831 | 1824 | 1824 | 1824 | 97.4 |
| JB_W_B2_M058 | 761 | 741 | 741 | 741 | 741 | 97.4 |
| JB_W_B2_M059 | 3300 | 3225 | 3217 | 3204 | 3204 | 97.1 |
| JB_W_B2_M060 | 574 | 563 | 563 | 563 | 563 | 98.1 |
| JB_W_B2_M061 | 1955 | 1906 | 1905 | 1905 | 1905 | 97.4 |
| JB_W_B2_M062 | 2796 | 2754 | 2753 | 2751 | 2751 | 98.4 |
| JB_W_B2_M063 | 3021 | 2957 | 2957 | 2957 | 2957 | 97.9 |
| JB_W_B2_M064 | 7859 | 7730 | 7728 | 7721 | 7721 | 98.2 |
| JB_W_B2_M097 | 2170 | 2136 | 2134 | 2126 | 2126 | 98 |
| JB_W_B2_M098 | 13883 | 13518 | 13513 | 13471 | 13451 | 96.9 |
| JB_W_B2_M099 | 16168 | 15822 | 15804 | 15764 | 15645 | 96.8 |
| JB_W_B2_M100 | 10849 | 10619 | 10607 | 10485 | 10439 | 96.2 |
| JB_W_B2_M101 | 1985 | 1925 | 1918 | 1871 | 1871 | 94.3 |
| JB_W_B2_M102 | 18498 | 17983 | 17980 | 17808 | 17808 | 96.3 |
| JB_W_B2_M103 | 4575 | 4475 | 4474 | 4465 | 4459 | 97.5 |
| JB_W_B2_M104 | 11270 | 10991 | 10991 | 10991 | 10991 | 97.5 |
| JB_W_B2_M105 | 2405 | 2266 | 2208 | 2200 | 2200 | 91.5 |
| JB_W_B2_M106 | 4392 | 4280 | 4280 | 4261 | 4261 | 97 |
| JB_W_B2_M107 | 8967 | 8777 | 8775 | 8727 | 8664 | 96.6 |
| JB_W_B2_M108 | 4436 | 4332 | 4327 | 4325 | 4325 | 97.5 |
| JB_W_B2_M109 | 10896 | 10640 | 10636 | 10601 | 10577 | 97.1 |
| JB_W_B2_M110 | 15392 | 15034 | 15028 | 14998 | 14975 | 97.3 |
| JB_W_B2_M111 | 3632 | 3551 | 3521 | 3517 | 3517 | 96.8 |
| JB_W_B2_M112 | 15751 | 15412 | 15402 | 15361 | 15308 | 97.2 |
| JB_W_B2_M137 | 11608 | 11421 | 11226 | 11139 | 11139 | 96 |
| JB_W_B2_M138 | 5408 | 5160 | 5159 | 5152 | 5152 | 95.3 |
| JB_W_B2_M139 | 29778 | 29181 | 29110 | 28878 | 28755 | 96.6 |
| JB_W_B2_M140 | 14806 | 14493 | 14490 | 14479 | 14449 | 97.6 |
| JB_W_B2_M141 | 5757 | 5644 | 5641 | 5636 | 5636 | 97.9 |
| JB_W_B2_M142 | 13024 | 12763 | 12758 | 12738 | 12738 | 97.8 |
| JB_W_B2_M143 | 1584 | 1545 | 1543 | 1540 | 1540 | 97.2 |
| JB_W_B2_M144 | 11487 | 11259 | 11240 | 11218 | 11197 | 97.5 |
| JB_W_B2_M153 | 10896 | 10708 | 10699 | 10670 | 10633 | 97.6 |
| JB_W_B2_M154 | 3751 | 3689 | 3680 | 3656 | 3656 | 97.5 |
| JB_W_B2_M155 | 5026 | 4947 | 4933 | 4900 | 4900 | 97.5 |
| JB_W_B2_M156 | 3835 | 3755 | 3753 | 3749 | 3749 | 97.8 |
| JB_W_B2_M157 | 752 | 714 | 708 | 707 | 707 | 94 |
| JB_W_B2_M158 | 7110 | 6962 | 6959 | 6956 | 6956 | 97.8 |
| JB_W_B2_M159 | 2425 | 2378 | 2378 | 2378 | 2378 | 98.1 |
| JB_W_B2_M160 | 8430 | 8268 | 8263 | 8222 | 8222 | 97.5 |
| JB_W_B2_M_NC_A3 | 4624 | 4524 | 4521 | 4509 | 4509 | 97.5 |
| JB_W_B2_M_NC_B3 | 983 | 962 | 957 | 957 | 957 | 97.4 |
| JB_W_B2_PCR1_C6 | 2539 | 2515 | 2515 | 2514 | 2514 | 99 |
| JB_W_B2_PCR1_D6 | 9188 | 9076 | 9076 | 8897 | 8897 | 96.8 |
| UME_W_B1_220_NC | 17192 | 16827 | 16815 | 16788 | 16551 | 96.3 |
| UME_W_B1_222_NC | 14783 | 14505 | 14490 | 14465 | 14347 | 97.1 |
| UME_W_B1_224_NC | 6487 | 6378 | 6372 | 6334 | 6281 | 96.8 |
| UME_W_B1_DNA_NC_A6 | 5188 | 5092 | 5085 | 5061 | 5028 | 96.9 |
| UME_W_B1_DNA_NC_B6 | 7826 | 7654 | 7649 | 7629 | 7592 | 97 |
| UME_W_B1_DNA_NC_G12 | 1144 | 1136 | 1131 | 1118 | 1118 | 97.7 |
| UME_W_B1_DNA_NC_H12 | 13189 | 12949 | 12944 | 12939 | 12939 | 98.1 |
| UME_W_B1_N001 | 14458 | 14223 | 14212 | 14145 | 13913 | 96.2 |
| UME_W_B1_N002 | 15541 | 15283 | 15267 | 15179 | 14890 | 95.8 |
| UME_W_B1_N003 | 21620 | 21236 | 21226 | 21110 | 20624 | 95.4 |
| UME_W_B1_N004 | 23061 | 22626 | 22594 | 22477 | 21896 | 94.9 |
| UME_W_B1_N005 | 5950 | 5477 | 5469 | 5413 | 5295 | 89 |
| UME_W_B1_N006 | 26266 | 25800 | 25750 | 25589 | 24836 | 94.6 |
| UME_W_B1_N007 | 16868 | 16607 | 16599 | 16548 | 16186 | 96 |
| UME_W_B1_N008 | 25356 | 24737 | 24675 | 24525 | 23706 | 93.5 |
| UME_W_B1_N025 | 26732 | 26182 | 26156 | 25983 | 25116 | 94 |
| UME_W_B1_N026 | 27588 | 27086 | 27062 | 26808 | 25965 | 94.1 |
| UME_W_B1_N027 | 21324 | 20952 | 20918 | 20750 | 20325 | 95.3 |
| UME_W_B1_N028 | 20919 | 20536 | 20506 | 20397 | 19779 | 94.6 |
| UME_W_B1_N029 | 16266 | 15965 | 15951 | 15821 | 15570 | 95.7 |
| UME_W_B1_N030 | 25810 | 25386 | 25376 | 25131 | 24643 | 95.5 |
| UME_W_B1_N031 | 14690 | 14395 | 14374 | 14271 | 14049 | 95.6 |
| UME_W_B1_N032 | 35178 | 34681 | 34511 | 34249 | 33257 | 94.5 |
| UME_W_B1_N033 | 9052 | 8885 | 8848 | 8725 | 8622 | 95.2 |
| UME_W_B1_N034 | 13377 | 13144 | 13129 | 12941 | 12736 | 95.2 |
| UME_W_B1_N035 | 15583 | 15308 | 15266 | 15155 | 14882 | 95.5 |
| UME_W_B1_N036 | 10229 | 10061 | 10016 | 9981 | 9856 | 96.4 |
| UME_W_B1_N037 | 5815 | 5722 | 5712 | 5681 | 5591 | 96.1 |
| UME_W_B1_N038 | 6314 | 6173 | 6162 | 6125 | 6032 | 95.5 |
| UME_W_B1_N039 | 9065 | 8870 | 8838 | 8685 | 8577 | 94.6 |
| UME_W_B1_N040 | 4019 | 3847 | 3820 | 3780 | 3748 | 93.3 |
| UME_W_B1_N049 | 6406 | 6195 | 6178 | 6128 | 6075 | 94.8 |
| UME_W_B1_N050 | 3952 | 3890 | 3883 | 3850 | 3818 | 96.6 |
| UME_W_B1_N051 | 14037 | 13797 | 13768 | 13695 | 13485 | 96.1 |
| UME_W_B1_N052 | 4028 | 3958 | 3946 | 3928 | 3897 | 96.7 |
| UME_W_B1_N053 | 5622 | 5516 | 5493 | 5473 | 5375 | 95.6 |
| UME_W_B1_N054 | 10868 | 10492 | 10472 | 10407 | 10312 | 94.9 |
| UME_W_B1_N055 | 7045 | 6901 | 6895 | 6843 | 6779 | 96.2 |
| UME_W_B1_N056 | 4321 | 4259 | 4249 | 4234 | 4192 | 97 |
| UME_W_B1_N057 | 30893 | 30331 | 30294 | 30015 | 29043 | 94 |
| UME_W_B1_N058 | 14188 | 13946 | 13899 | 13707 | 13551 | 95.5 |
| UME_W_B1_N059 | 16325 | 16032 | 15928 | 15703 | 15119 | 92.6 |
| UME_W_B1_N060 | 13551 | 13279 | 13267 | 13221 | 13006 | 96 |
| UME_W_B1_N061 | 11531 | 11261 | 11220 | 11135 | 10919 | 94.7 |
| UME_W_B1_N062 | 14445 | 14174 | 14153 | 14050 | 13866 | 96 |
| UME_W_B1_N063 | 29253 | 28759 | 28730 | 28525 | 27818 | 95.1 |
| UME_W_B1_N064 | 15332 | 15098 | 15069 | 14957 | 14600 | 95.2 |
| UME_W_B1_N089 | 4219 | 4136 | 4134 | 4119 | 4083 | 96.8 |
| UME_W_B1_N090 | 3991 | 3926 | 3839 | 3792 | 3785 | 94.8 |
| UME_W_B1_N091 | 11711 | 11520 | 11511 | 11410 | 11300 | 96.5 |
| UME_W_B1_N092 | 2339 | 2287 | 2283 | 2259 | 2259 | 96.6 |
| UME_W_B1_N093 | 10607 | 10409 | 10403 | 10320 | 10220 | 96.4 |
| UME_W_B1_N094 | 7923 | 7774 | 7761 | 7687 | 7667 | 96.8 |
| UME_W_B1_N095 | 3145 | 2141 | 2119 | 2094 | 2094 | 66.6 |
| UME_W_B1_N096 | 2651 | 2585 | 2576 | 2560 | 2550 | 96.2 |
| UME_W_B1_N097 | 34396 | 33708 | 33701 | 33591 | 33108 | 96.3 |
| UME_W_B1_N098 | 8415 | 8235 | 8214 | 8131 | 8046 | 95.6 |
| UME_W_B1_N099 | 6109 | 6017 | 5998 | 5844 | 5778 | 94.6 |
| UME_W_B1_N100 | 35795 | 35192 | 35155 | 34919 | 33506 | 93.6 |
| UME_W_B1_N125 | 6023 | 5911 | 5895 | 5818 | 5799 | 96.3 |
| UME_W_B1_N127 | 15674 | 15381 | 15358 | 15232 | 14967 | 95.5 |
| UME_W_B1_N128 | 2172 | 2132 | 2112 | 2104 | 2089 | 96.2 |
| UME_W_B1_N129 | 1726 | 1674 | 1661 | 1646 | 1636 | 94.8 |
| UME_W_B1_N130 | 3913 | 3844 | 3802 | 3774 | 3733 | 95.4 |
| UME_W_B1_N131 | 2242 | 2176 | 2153 | 2130 | 2130 | 95 |
| UME_W_B1_N132 | 1382 | 1349 | 1336 | 1329 | 1329 | 96.2 |
| UME_W_B1_N133 | 11815 | 11612 | 11583 | 11411 | 11169 | 94.5 |
| UME_W_B1_N134 | 29991 | 29475 | 29465 | 29284 | 28502 | 95 |
| UME_W_B1_N135 | 6224 | 6113 | 6100 | 6065 | 5942 | 95.5 |
| UME_W_B1_N136 | 13479 | 13219 | 13198 | 13018 | 12748 | 94.6 |
| UME_W_B1_N137 | 8038 | 7864 | 7855 | 7748 | 7642 | 95.1 |
| UME_W_B1_N138 | 4147 | 4071 | 4060 | 4027 | 3987 | 96.1 |
| UME_W_B1_N139 | 14578 | 14226 | 14211 | 14094 | 13874 | 95.2 |
| UME_W_B1_N140 | 8118 | 7992 | 7944 | 7799 | 7676 | 94.6 |
| UME_W_B1_N142 | 13683 | 13448 | 13436 | 13196 | 12864 | 94 |
| UME_W_B1_N143 | 19962 | 19652 | 19642 | 19601 | 19346 | 96.9 |
| UME_W_B1_N144 | 30733 | 30223 | 30125 | 29916 | 29618 | 96.4 |
| UME_W_B1_N145 | 25091 | 24648 | 24597 | 24216 | 23497 | 93.6 |
| UME_W_B1_N146 | 14903 | 14654 | 14637 | 14448 | 14139 | 94.9 |
| UME_W_B1_N147 | 12343 | 12148 | 12138 | 11985 | 11782 | 95.5 |
| UME_W_B1_N148 | 30934 | 30441 | 30403 | 30104 | 29214 | 94.4 |
| UME_W_B1_PCR1_C6 | 481 | 477 | 477 | 477 | 477 | 99.2 |
| UME_W_B1_PCR1_D6 | 677 | 668 | 668 | 668 | 668 | 98.7 |
| UME_W_B1_PCR1_F12 | 448 | 432 | 432 | 432 | 432 | 96.4 |
| UME_W_B2_221_NC | 15765 | 15502 | 15489 | 15455 | 15374 | 97.5 |
| UME_W_B2_223_NC | 16266 | 15973 | 15957 | 15901 | 15783 | 97 |
| UME_W_B2_226_NC | 29138 | 28664 | 28629 | 28395 | 28117 | 96.5 |
| UME_W_B2_DNA_NC_A6 | 32001 | 31417 | 31384 | 31241 | 30943 | 96.7 |
| UME_W_B2_DNA_NC_B6 | 10285 | 10130 | 10119 | 9982 | 9906 | 96.3 |
| UME_W_B2_DNA_NC_G12 | 4897 | 4848 | 4848 | 4833 | 4833 | 98.7 |
| UME_W_B2_DNA_NC_H12 | 10923 | 10704 | 10702 | 10664 | 10664 | 97.6 |
| UME_W_B2_N009 | 10191 | 9982 | 9962 | 9907 | 9698 | 95.2 |
| UME_W_B2_N010 | 29111 | 28683 | 28652 | 28497 | 27684 | 95.1 |
| UME_W_B2_N011 | 41431 | 40764 | 40735 | 40442 | 39391 | 95.1 |
| UME_W_B2_N012 | 17769 | 17395 | 17372 | 17303 | 16959 | 95.4 |
| UME_W_B2_N013 | 13334 | 12676 | 12653 | 12639 | 12237 | 91.8 |
| UME_W_B2_N014 | 26735 | 26272 | 26257 | 26147 | 25533 | 95.5 |
| UME_W_B2_N015 | 26955 | 26544 | 26522 | 26321 | 25565 | 94.8 |
| UME_W_B2_N016 | 17569 | 17279 | 17270 | 17181 | 16807 | 95.7 |
| UME_W_B2_N017 | 7979 | 7795 | 7783 | 7748 | 7710 | 96.6 |
| UME_W_B2_N018 | 11191 | 9911 | 9897 | 9856 | 9766 | 87.3 |
| UME_W_B2_N019 | 12691 | 12452 | 12439 | 12395 | 12181 | 96 |
| UME_W_B2_N020 | 19754 | 19401 | 19352 | 19288 | 18998 | 96.2 |
| UME_W_B2_N021 | 24567 | 24217 | 24188 | 24031 | 23577 | 96 |
| UME_W_B2_N022 | 3954 | 3850 | 3830 | 3809 | 3778 | 95.5 |
| UME_W_B2_N023 | 4533 | 4466 | 4451 | 4439 | 4415 | 97.4 |
| UME_W_B2_N024 | 13667 | 13156 | 13131 | 13019 | 12689 | 92.8 |
| UME_W_B2_N041 | 25443 | 24927 | 24885 | 24798 | 24167 | 95 |
| UME_W_B2_N042 | 10069 | 9909 | 9903 | 9882 | 9829 | 97.6 |
| UME_W_B2_N043 | 5425 | 5327 | 5320 | 5309 | 5302 | 97.7 |
| UME_W_B2_N044 | 4068 | 4001 | 3993 | 3983 | 3983 | 97.9 |
| UME_W_B2_N045 | 13541 | 13326 | 13312 | 13201 | 13082 | 96.6 |
| UME_W_B2_N046 | 8504 | 8371 | 8366 | 8292 | 8236 | 96.8 |
| UME_W_B2_N047 | 7442 | 7329 | 7311 | 7239 | 7183 | 96.5 |
| UME_W_B2_N048 | 11750 | 11532 | 11505 | 11444 | 11312 | 96.3 |
| UME_W_B2_N065 | 32076 | 31513 | 31470 | 31333 | 30156 | 94 |
| UME_W_B2_N066 | 48199 | 47404 | 47391 | 47210 | 46219 | 95.9 |
| UME_W_B2_N067 | 32282 | 31670 | 31577 | 31301 | 29818 | 92.4 |
| UME_W_B2_N068 | 45037 | 44042 | 43986 | 43611 | 42107 | 93.5 |
| UME_W_B2_N069 | 26142 | 24071 | 24039 | 23839 | 23224 | 88.8 |
| UME_W_B2_N070 | 11524 | 11341 | 11333 | 11217 | 11000 | 95.5 |
| UME_W_B2_N071 | 44494 | 43644 | 43605 | 43287 | 42060 | 94.5 |
| UME_W_B2_N072 | 23519 | 23128 | 23121 | 22900 | 22036 | 93.7 |
| UME_W_B2_N073 | 4038 | 3952 | 3935 | 3916 | 3893 | 96.4 |
| UME_W_B2_N074 | 5314 | 5213 | 5194 | 5182 | 5151 | 96.9 |
| UME_W_B2_N075 | 2967 | 2910 | 2908 | 2896 | 2896 | 97.6 |
| UME_W_B2_N076 | 14521 | 14241 | 14224 | 14150 | 13918 | 95.8 |
| UME_W_B2_N077 | 972 | 936 | 932 | 929 | 929 | 95.6 |
| UME_W_B2_N078 | 4261 | 4154 | 4132 | 4112 | 4112 | 96.5 |
| UME_W_B2_N079 | 1628 | 1582 | 1581 | 1579 | 1579 | 97 |
| UME_W_B2_N080 | 5172 | 5078 | 5024 | 4993 | 4993 | 96.5 |
| UME_W_B2_N081 | 22631 | 22239 | 22179 | 21889 | 21420 | 94.6 |
| UME_W_B2_N082 | 18146 | 17900 | 17823 | 17642 | 17389 | 95.8 |
| UME_W_B2_N083 | 20818 | 20438 | 20412 | 20193 | 19618 | 94.2 |
| UME_W_B2_N084 | 23012 | 22515 | 22484 | 22225 | 21756 | 94.5 |
| UME_W_B2_N085 | 9084 | 8932 | 8911 | 8832 | 8751 | 96.3 |
| UME_W_B2_N086 | 16519 | 16177 | 16170 | 16086 | 15861 | 96 |
| UME_W_B2_N087 | 7700 | 7589 | 7563 | 7493 | 7468 | 97 |
| UME_W_B2_N088 | 24555 | 24143 | 24109 | 23864 | 22821 | 92.9 |
| UME_W_B2_N101 | 34416 | 33825 | 33811 | 33653 | 32866 | 95.5 |
| UME_W_B2_N102 | 6937 | 6815 | 6801 | 6738 | 6674 | 96.2 |
| UME_W_B2_N103 | 8825 | 8688 | 8671 | 8603 | 8571 | 97.1 |
| UME_W_B2_N104 | 45426 | 44566 | 44553 | 44288 | 43135 | 95 |
| UME_W_B2_N105 | 8112 | 7975 | 7957 | 7908 | 7790 | 96 |
| UME_W_B2_N106 | 11182 | 10999 | 10990 | 10956 | 10902 | 97.5 |
| UME_W_B2_N107 | 53330 | 52246 | 52233 | 51979 | 50536 | 94.8 |
| UME_W_B2_N108 | 19902 | 19506 | 19481 | 19298 | 18627 | 93.6 |
| UME_W_B2_N109 | 12248 | 12022 | 12005 | 11918 | 11803 | 96.4 |
| UME_W_B2_N110 | 4881 | 4800 | 4792 | 4735 | 4722 | 96.7 |
| UME_W_B2_N111 | 9796 | 9637 | 9629 | 9411 | 9333 | 95.3 |
| UME_W_B2_N112 | 32491 | 31876 | 31853 | 31588 | 31254 | 96.2 |
| UME_W_B2_N113 | 8254 | 8062 | 8055 | 7993 | 7993 | 96.8 |
| UME_W_B2_N114 | 20870 | 18296 | 18281 | 18035 | 17843 | 85.5 |
| UME_W_B2_N115 | 14185 | 13958 | 13933 | 13840 | 13686 | 96.5 |
| UME_W_B2_N116 | 14260 | 13764 | 13733 | 13613 | 13302 | 93.3 |
| UME_W_B2_N117 | 22597 | 22133 | 22098 | 22035 | 21679 | 95.9 |
| UME_W_B2_N118 | 5094 | 5002 | 4995 | 4980 | 4980 | 97.8 |
| UME_W_B2_N119 | 14599 | 14362 | 14347 | 14170 | 14030 | 96.1 |
| UME_W_B2_N120 | 4018 | 3927 | 3923 | 3894 | 3894 | 96.9 |
| UME_W_B2_N121 | 24305 | 23903 | 23840 | 23589 | 23262 | 95.7 |
| UME_W_B2_N122 | 5427 | 5246 | 5242 | 5188 | 5188 | 95.6 |
| UME_W_B2_N123 | 1698 | 1680 | 1672 | 1661 | 1661 | 97.8 |
| UME_W_B2_N124 | 3926 | 3842 | 3836 | 3809 | 3809 | 97 |
| UME_W_B2_N149 | 11603 | 11441 | 11437 | 11386 | 11305 | 97.4 |
| UME_W_B2_N150 | 15291 | 14778 | 14721 | 14674 | 14472 | 94.6 |
| UME_W_B2_N151 | 19626 | 19387 | 19366 | 19184 | 18666 | 95.1 |
| UME_W_B2_N152 | 28360 | 27838 | 27780 | 27526 | 26901 | 94.9 |

**References**

1. Caporaso JG, Lauber CL, Walters WA, Berg-Lyons D, Huntley J, Fierer N, Owens SM, Betley J, Fraser L, Bauer M, Gormley N, Gilbert JA, Smith G, Knight R (2012) Ultra-high-throughput microbial community analysis on the Illumina HiSeq and MiSeq platforms. ISME J 6: 1621-1624. doi: 10.1038/ismej.2012.8

2. Apprill A, McNally S, Parsons R, Weber L (2015) Minor revision to V4 region SSU rRNA 806R gene primer greatly increases detection of SAR11 bacterioplankton. Aquat Microb Ecol 75: 129-137.

3. Parada AE, Needham DM, Fuhrman JA (2016) Every base matters: assessing small subunit rRNA primers for marine microbiomes with mock communities, time series and global field samples. Environ Microbiol 18: 1403-1414. doi: <https://doi.org/10.1111/1462-2920.13023>

4. Ritalahti KM, Amos BK, Sung Y, Wu Q, Koenigsberg SS, Löffler FE (2006) Quantitative PCR targeting 16S rRNA and reductive dehalogenase genes simultaneously monitors multiple *Dehalococcoides* strains. Appl Environ Microbiol 72: 2765-2774.

5. Martin M (2011) Cutadapt removes adapter sequences from high-throughput sequencing reads. EMBnet J 17: 10-12. doi: 10.14806/ej.17.1.200

6. Callahan BJ, McMurdie PJ, Rosen MJ, Han AW, Johnson AJA, Holmes SP (2016) DADA2: High-resolution sample inference from Illumina amplicon data. Nat Methods 13: 581-583. doi: 10.1038/nmeth.3869

7. Yilmaz P, Parfrey LW, Yarza P, Gerken J, Pruesse E, Quast C, Schweer T, Peplies J, Ludwig W, Glöckner FO (2014) The SILVA and “all-species living tree project (LTP)” taxonomic frameworks. Nucleic Acids Res 42: D643-D648.

8. McMurdie PJ, Holmes S (2013) phyloseq: An R package for reproducible interactive analysis and graphics of microbiome census data. PLoS One 8: e61217. doi: 10.1371/journal.pone.0061217

9. Hsieh TC, Ma KH, Chao A (2016) iNEXT: an R package for rarefaction and extrapolation of species diversity (Hill numbers). Methods in Ecology and Evolution 7: 1451-1456. doi: <https://doi.org/10.1111/2041-210X.12613>

10. Schloss PD (2021) Amplicon sequence variants artificially split bacterial genomes into separate clusters. mSphere 6: 10.1128/msphere.00191-00121. doi: doi:10.1128/msphere.00191-21

11. McCauley M, Goulet TL, Jackson CR, Loesgen S (2023) Systematic review of cnidarian microbiomes reveals insights into the structure, specificity, and fidelity of marine associations. Nat Commun 14: 4899. doi: 10.1038/s41467-023-39876-6

12. Wright ES (2016) Using DECIPHER v2. 0 to analyze big biological sequence data in R. R J 8.

13. McLaren M (2024) speedyseq: Faster implementations of common phyloseq functions.

14. Tamarit D, Ellegaard KM, Wikander J, Olofsson T, Vásquez A, Andersson SGE (2015) Functionally structured genomes in *Lactobacillus kunkeei* colonizing the honey crop and food products of honeybees and stingless bees. Genome Biol Evol 7: 1455-1473. doi: 10.1093/gbe/evv079

15. Ogier J-C, Pagès S, Galan M, Barret M, Gaudriault S (2019) *rpoB*, a promising marker for analyzing the diversity of bacterial communities by amplicon sequencing. BMC Microbiol 19: 171. doi: 10.1186/s12866-019-1546-z

16. Dyrhage K, Garcia-Montaner A, Tamarit D, Seeger C, Näslund K, Olofsson TC, Vasquez A, Webster MT, Andersson SG (2022) Genome evolution of a symbiont population for pathogen defense in honeybees. Genome Biol Evol 14: evac153.

17. Katoh K, Standley DM (2013) MAFFT Multiple sequence alignment software version 7: improvements in performance and usability. Mol Biol Evol 30: 772-780. doi: 10.1093/molbev/mst010

18. Capella-Gutiérrez S, Silla-Martínez JM, Gabaldón T (2009) trimAl: a tool for automated alignment trimming in large-scale phylogenetic analyses. Bioinformatics 25: 1972-1973.

19. Minh BQ, Schmidt HA, Chernomor O, Schrempf D, Woodhams MD, von Haeseler A, Lanfear R (2020) IQ-TREE 2: new models and efficient methods for phylogenetic inference in the genomic era. Mol Biol Evol 37: 1530-1534. doi: 10.1093/molbev/msaa015

20. Anisimova M, Gil M, Dufayard J-F, Dessimoz C, Gascuel O (2011) Survey of branch support methods demonstrates accuracy, power, and robustness of fast likelihood-based approximation schemes. Syst Biol 60: 685-699. doi: 10.1093/sysbio/syr041

21. Hoang DT, Chernomor O, von Haeseler A, Minh BQ, Vinh LS (2017) UFBoot2: improving the ultrafast bootstrap approximation. Mol Biol Evol 35: 518-522. doi: 10.1093/molbev/msx281

22. Kalyaanamoorthy S, Minh BQ, Wong TKF, von Haeseler A, Jermiin LS (2017) ModelFinder: fast model selection for accurate phylogenetic estimates. Nat Methods 14: 587-589. doi: 10.1038/nmeth.4285

23. Mai U, Mirarab S (2018) TreeShrink: fast and accurate detection of outlier long branches in collections of phylogenetic trees. BMC Genomics 19: 23-40.
